# Role of Tertiary Lymphoid Structures in Autoimmune Diseases and Cancer

**DOI:** 10.1002/mco2.70943

**Published:** 2026-09-18

**Authors:** Ze Yan, Bin Wen, Ying Hu, Xiaocui Wang, Xuemei Duan, Haonan Li, Shumin Dong, Yufeng Fan, Caihong Wang, Shutong Yang, Huifeng Shang, Yukai Jing

**Affiliations:** ^1^ School of Public Health Shanxi Medical University Taiyuan China; ^2^ Department of Clinical Laboratory Third Hospital of Shanxi Medical University Shanxi Bethune Hospital Shanxi Academy of Medical Sciences Tongji Shanxi Hospital Taiyuan China

**Keywords:** autoimmune diseases, cancer, immune cells, immune response, tertiary lymphoid structures

## Abstract

Tertiary lymphoid structures (TLSs) are ectopic, organized immune aggregates forming in non‐lymphoid tissues during chronic inflammation. Resembling secondary lymphoid organs (SLOs) architecturally and functionally, TLSs feature specialized stromal networks and compartmentalized immune cell zones. In autoimmunity, TLSs can perpetuate local immune activation, autoantibody production, and tissue damage, correlating with disease severity and progression. In the tumor microenvironment, TLSs are generally associated with enhanced anti‐tumor immunity, improved patient survival, and better responses to immunotherapy, although their functional impact can vary across cancer types. This review systematically examines the structural and functional foundations of TLSs and dissects the mechanisms driving their formation in both autoimmune disorders and cancers. We critically evaluate the clinical correlations of TLSs with disease activity, progression, and patient outcomes. Furthermore, we summarize current methodologies for TLS detection and assess their diagnostic and prognostic utility in disease monitoring. Finally, we discuss emerging therapeutic strategies aimed at modulating TLSs formation or function. By synthesizing current knowledge, this review provides crucial insights into TLSs biology within autoimmunity and cancer, highlighting their potential as novel biomarkers and therapeutic targets, thereby advancing our understanding of disease mechanisms and treatment approaches.

## Graphical abstract

Schematic overview of tertiary lymphoid structures (TLSs): The development process, detection, and the immune function that depends on the environment in different disease contexts of TLS. TLSs originate from loose lymphoid aggregates that acquire ordered follicular architecture to become primary follicular‐like TLS, before maturing into compartmentalized immune hubs with segregated T and B cell zones, intact follicular dendritic cell networks and functional high endothelial venules. Routine histopathological staining, multiplex immune labeling, medical imaging and machine learning–assisted analysis constitute the primary modalities for TLS identification, with these detection tools supporting diagnostic stratification, immunotherapy regimen adjustment, and individualized patient treatment design. Pro‐inflammatory chemokine and cytokine cascades originating from TLSs amplify autoreactive immune responses and drive tissue injury in autoimmune conditions including SLE, RA, and MS. TLS function as a “double‐edged sword” within the tumor microenvironment, their dual effects dictated by intratumoral localization, cellular maturation status, and local stromal cues: mature intratumoral TLSs equipped with germinal centers sustain potent anti‐tumor immunity, whereas sparse, immature peritumoral TLSs recruit exhausted T cells to form immunosuppressive niches, which drive poor clinical outcomes in malignancies including HGSOC, HNSCC, and CRC. This image was created by Figdraw.

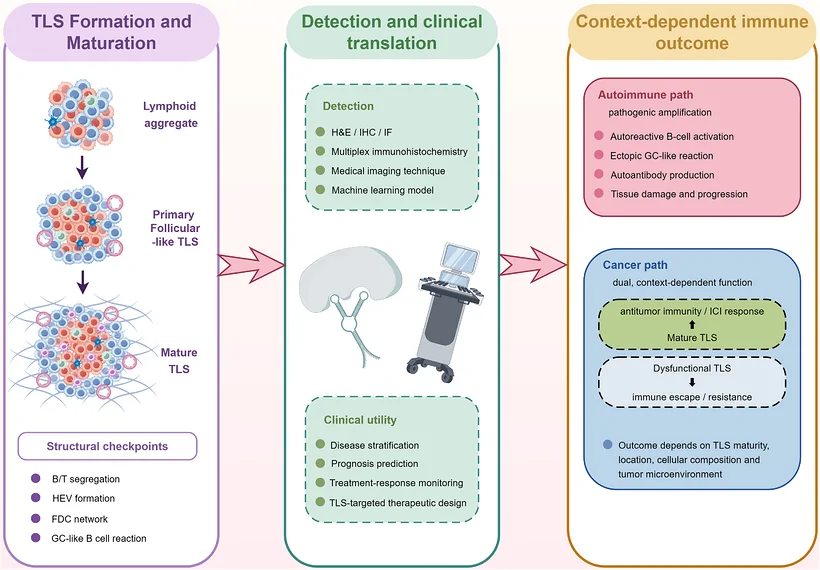

## 1 Introduction

Autoimmune diseases and cancer are two major chronic diseases worldwide, and both are driven by chronic inflammation. Cancer affects over 20 million people worldwide annually, and inflammation is involved in 15%–20% of cases [1]. A key shared feature is tertiary lymphoid structures (TLSs)—organized immune aggregates that form in inflamed tissues outside normal lymphoid organs. In autoimmune diseases, TLSs appear in damaged organs (e.g., SLE kidneys, RA synovium); in cancer, they form inside or around tumors (e.g., lung cancer, colorectal cancer) [2, 3]. TLSs are found in both autoimmune diseases and cancer, but their role depends on the context: in conditions requiring enhanced immune responses, such as cancer and infectious diseases, the presence and maturation of TLSs generally correlate with better clinical outcomes, reflecting their capacity to support effective antitumor or antipathogen immunity [2, 4, 5, 6, 7, 8]. Conversely, in autoimmune diseases and age‐related chronic inflammatory diseases, where the immune system abnormally targets self‐tissues, the appearance and maturation of TLSs are frequently associated with poorer outcomes, as these structures sustain pathogenic autoimmune activation and exacerbate tissue damage [9, 10, 11]. Therefore, TLSs can be seen as a double‐edged sword. This functional duality, combined with their potential as biomarkers and therapeutic targets, highlights the need to synthesize TLS biology in the field of autoimmune diseases and cancer.

Autoimmune disorders encompass a heterogeneous group of conditions, including systemic lupus erythematosus (SLE) [12], rheumatoid arthritis (RA) [13, 14], multiple sclerosis (MS) [15, 16], type I diabetes mellitus (TID) [17, 18, 19], and inflammatory bowel disease (IBD) [20, 21]. Recent advancement in biomedical research has substantially enhanced our understanding of autoimmune pathophysiology. Afflicting approximately 5% of the global population, these diseases impose profound physical, psychological, and socioeconomic burdens on patients, with many remaining refractory to curative therapies [22]. The public health impact of autoimmune disorders is quantifiable through three key epidemiological metrics: incidence rates, mortality rates, and years of life lost (YLL). Globally, autoimmune disease rates are rising—TID incidence has risen by 3%–4% annually over the past 30 years [23]. SLE and RA affect women of reproductive age hardest, often damaging multiple organs [24]. Mortality analyses reveal elevated risks in autoimmune populations: systemic autoimmune diseases (SAIDs) demonstrate mortality rates of 14.6 per million population as underlying causes of death (CoD) and 39.7 for any‐mention CoD [23]. Premature mortality further exacerbates disease burden, SLE patients died 6 years earlier than matched controls on average, with 17.3% succumbing before 60 years old [23, 24]. Even non‐fatal outcomes carry significant consequences: some autoimmune encephalitis (AE) patients have lasting brain issues that diminish health‐related quality of life (HRQOL) and keep them from work or school [25].

Autoimmune pathogenesis involves aberrant immune activation triggered by diverse stimuli, including chronic inflammation, neoplastic processes, and allograft rejection [26]. This dysregulation culminates in immune‐mediated tissue damage and organ dysfunction. Within chronically inflamed non‐lymphoid tissues, TLSs—also termed ectopic lymphoid organs—develop as organized immune aggregates supported by specialized fibroblastic networks [27, 28]. These structures are characterized by organized lymphoid aggregates supported by a specialized fibroblast network, and they have many functional and structural characteristics in common with SLOs (Table 1). As an illustration, TLSs and SLOs promote immune responses that are specific to antigens and are identified by specialized blood vessels known as high endothelial venules (HEVs), which facilitate the movement of lymphocytes from the blood stream into lymphoid tissues [29]. In autoimmune diseases, TLSs coordinates localized immune responses through resident autoreactive T/B cells, driving tissue damage (e.g., autoantibody production in SLE renal TLSs) [30, 31, 32]; in cancer, TLSs recruit and activate anti‐tumor immune cells, such as cytotoxic CD8^+^ T cells [33, 34], and B cells to produce tumor‐specific antibodies, which correlate with improved prognosis and immunotherapy response in most solid cancers [35, 36]. TLSs emerge across multiple

**TABLE 1:** Comparative features of TLSs and SLOs.

Feature	Similarities	Differences	Refs.
Developmental trigger	Both depend on reciprocal lymphocyte‐stromal cell interactions for development and maintenance.	**SLOs**: Embryonic program (tissue‐specific)	[28, 37, 38]
		**TLSs**: Postnatal inflammation‐driven (no anatomical specificity)	
Core cellular players	Both rely on lymphoid Tissue inducer (LTi) cells and lymphoid Tissue organizer (LTo) stromal cells.	**LTi origin—SLOs**: Fetal liver‐derived	[39]
		**LTi origin—TLSs**: Adult hematopoietic precursors	
Molecular mechanisms	Both utilize LTα1β2‐LTβR signaling essential and chemokines (CXCL13, CCL19/21) drive organization for structural organization.	**SLOs**: Constitutive chemokine expression	[40, 41]
		**TLSs**: Inflammation‐induced chemokine production	
Structural organization	Both develop segregated T/B cell zones, HEVs, and FDCs in mature structures.	**SLOs**: Always encapsulated and afferent lymphatics present	[37, 40]
		**TLSs**: Never encapsulated and no afferent lymphatics	
Maturation process	Both undergo progressive organization from immature to functionally mature structures.	**SLOs**: Fixed developmental timeline (embryonic)	[42, 43]
		**TLSs**: Dynamic 3‐stage maturation:	
		1. Lymphocyte aggregates (no zones/FDCs)	
		2. Segregated T/B zones (no GCs)	
		3. Mature TLS with GCs/FDCs/HEVs	
Functional output	Both support adaptive immunity and facilitate antigen presentation.	**SLOs**: Systemic immunity/tolerance	[42]
		**TLSs**: Local autoimmunity (exposed to tissue antigens)	

 chronic inflammatory contexts, particularly autoimmune diseases and cancer [29, 35, 44, 45], where they orchestrate localized immune responses through resident immune cell populations or conserved molecular pathways [37, 46]. Their functional duality, potentially protective or pathogenic, depending on disease context, positions TLSs as promising targets for novel immunomodulatory therapies and combination treatment paradigms [47].

Despite the fact that there are some advances in TLS‐related research, the precise function of TLSs in autoimmune diseases and cancer remains inadequately elucidated. In autoimmune diseases, which involve the immune system attacking self‐tissues, the emergence and maturation of TLSs are often associated with poorer prognoses [10, 11, 48]; in cancer, mature TLSs (mTLSs) with germinal centers (GCs) typically correlate with good prognosis in most cancer types, but their presence has been linked to tumor development or progression in some cancer types [49, 50, 51]. Therefore, TLSs can be seen as a double‐edged sword. To fully harness the potential of TLSs, a comprehensive understanding of TLSs is urgently needed.

This review systematically includes three aspects: analyzing the functional and structural hallmarks of TLSs in autoimmune diseases and cancer and summarizing the detection methods for TLSs. Furthermore, we elucidate the mechanistic pathways underlying their formation in autoimmune diseases and cancer, and evaluate their clinical correlations with specific autoimmune conditions and cancer. In addition, we critically appraise emerging evidence regarding TLS‐mediated modulation of diagnostic biomarkers, while proposing a translational framework that integrates TLS‐targeted therapeutic strategies with precision monitoring protocols.

## 2 Structural and Functional Basis of TLSs

### 2.1 Formation of TLSs

TLSs are ectopic lymphoid formations that develop in non‐lymphoid organs under chronic inflammatory conditions [52]. Similar to SLOs, the formation of TLSs is initiated by the homing of lymphoid tissue inducer (LTi) cells or their functional substitute cells to sites of persistent inflammation [37, 53, 54]. A variety of sustained antigenic stimuli, including microbial agents, viruses, tobacco exposure, and certain drugs, can drive local production of pro‐inflammatory mediators, which in turn promote the recruitment and activation of these inducer cells [55, 56, 57, 58]. Subsequently, the activated LTi cells interact with resident stromal lymphoid tissue organizer (LTo) cells. This interaction activates key signaling pathways such as lymphotoxin α1β2/lymphotoxin beta receptor (LTα1β2/LTβR), interleukin‐7/interleukin‐7 receptor (IL‐7/IL‐7R), IL‐17/IL‐17R, and RANK/RANKL, thereby triggering the expression of chemokines and adhesion molecules that are essential for the coordinated recruitment and organization of lymphocytes, ultimately leading to TLS maturation [59, 60, 61]. Furthermore, these cytokines regulate cellular activities associated with TLS maturation at different stages and recruit additional lymphocytes and dendritic cells. This process is further regulated by other cytokines and requires both persistent antigen stimulation and inflammatory signals in the tissue microenvironment.

The maturation of TLSs is a dynamic, multi‐stage process. The earliest “precursor” stage consists of loose lymphocyte aggregates, which are characterized by dense clusters of lymphocytes within stromal fibroblasts, but have not yet developed distinct T/B cell zones or follicular dendritic cells (FDCs). As the tissue progresses, these aggregates develop into early (or primary follicular‐like) TLSs. Here, T cells and B cells start to separate into discernible areas, but a functional GC response has not yet formed. The final stage produces mTLSs, which are fully organized, featuring a clear T‐cell zone and a B‐cell follicle containing an active GC and a supporting network of FDCs. Notably, throughout these stages, a key feature is the gradual formation of specialized HEVs, which allows lymphocytes to be steadily recruited from the bloodstream.

### 2.2 Structural Composition of TLSs

**FIGURE 1:**
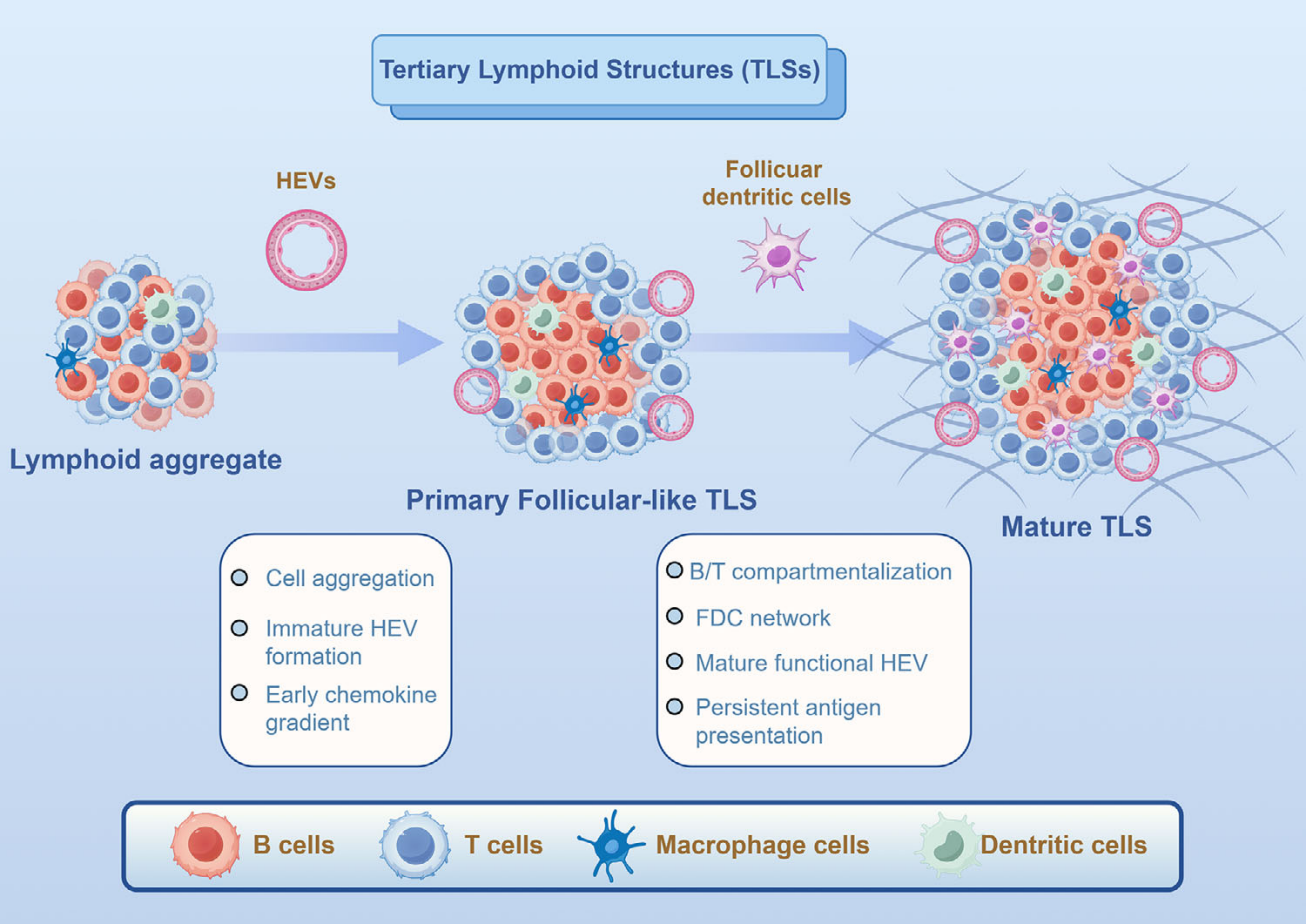
Schematic diagram of TLS maturation stages. Three successive developmental stages are linked by directional arrows, with text boxes marking defining features of each phase. Early lymphoid aggregates feature random immune cell aggregation, immature HEVs and preliminary chemokine gradients. Further cellular rearrangement produces primary follicular‐like TLS with preliminary T/B segregation. Fully mature TLS gain complete B/T compartmentalization, intact FDC networks, functional mature HEVs and sustained local antigen presentation. This image was created by Figdraw.

Architecturally, mTLSs exhibit a highly organized structure that mirrors SLOs. They consist of two fundamental components: a cellular compartment and an organizing extracellular matrix (ECM). The cellular compartment consists of lymphocytes, fibroblastic reticular cells (FRCs) [62], antigen‐presenting cells (APCs) [37], and HEVs [63]. The ECM is enriched with collagen (providing structural stability), fibronectin (mediating cell adhesion and migration), and signaling molecules [64, 65]. Histologically, mTLSs exhibit compartmentalized architecture: a B cell‐rich core containing GCs, surrounded by a T cell zone at the periphery, and are composed of both immune and stromal cells [28, 37]. The B cell domain integrates FDCs and macrophages, while the T cell region features mature DCs and T cells (Figure 1).

### 2.3 Function and Resolution of TLSs

Functionally, TLSs establish specialized microenvironments that recapitulate key immunological functions of SLOs. These include: coordinated immune cell activation and proliferation via antigen presentation [66, 67]; targeted immune cell recruitment through chemokine gradients, particularly CXCL13 and CCL21, which mediate lymphocyte and DC trafficking [45, 68, 69]; and localized autoantibody production in autoimmune pathologies, as evidenced by disease‐specific antibody titers within TLS‐containing tissues [70, 71].

TLSs are dynamic structures. Their resolution or persistence is tightly linked to the inflammatory context. Upon antigen clearance and resolution of inflammation, the chemokine gradients that sustain TLSs dissipate, leading to the end of lymphocyte recruitment, cellular apoptosis, and stromal remodeling, ultimately resulting in TLS disappearance [72, 73]. This reversible nature is shown by the fact that TLSs regress after immunosuppressive therapies, including corticosteroids [74, 75, 76]. Conversely, TLSs may be maintained indefinitely in chronic conditions where antigenic stimulation persists, such as in autoimmune flares or established tumors. In some pathological contexts, this chronicity can lead to dysfunction, where TLSs may foster immune tolerance or support a pro‐inflammatory niche that exacerbates pathology, highlighting their dual and context‐dependent role.

## 3 The Detection of TLSs

**FIGURE 2:**
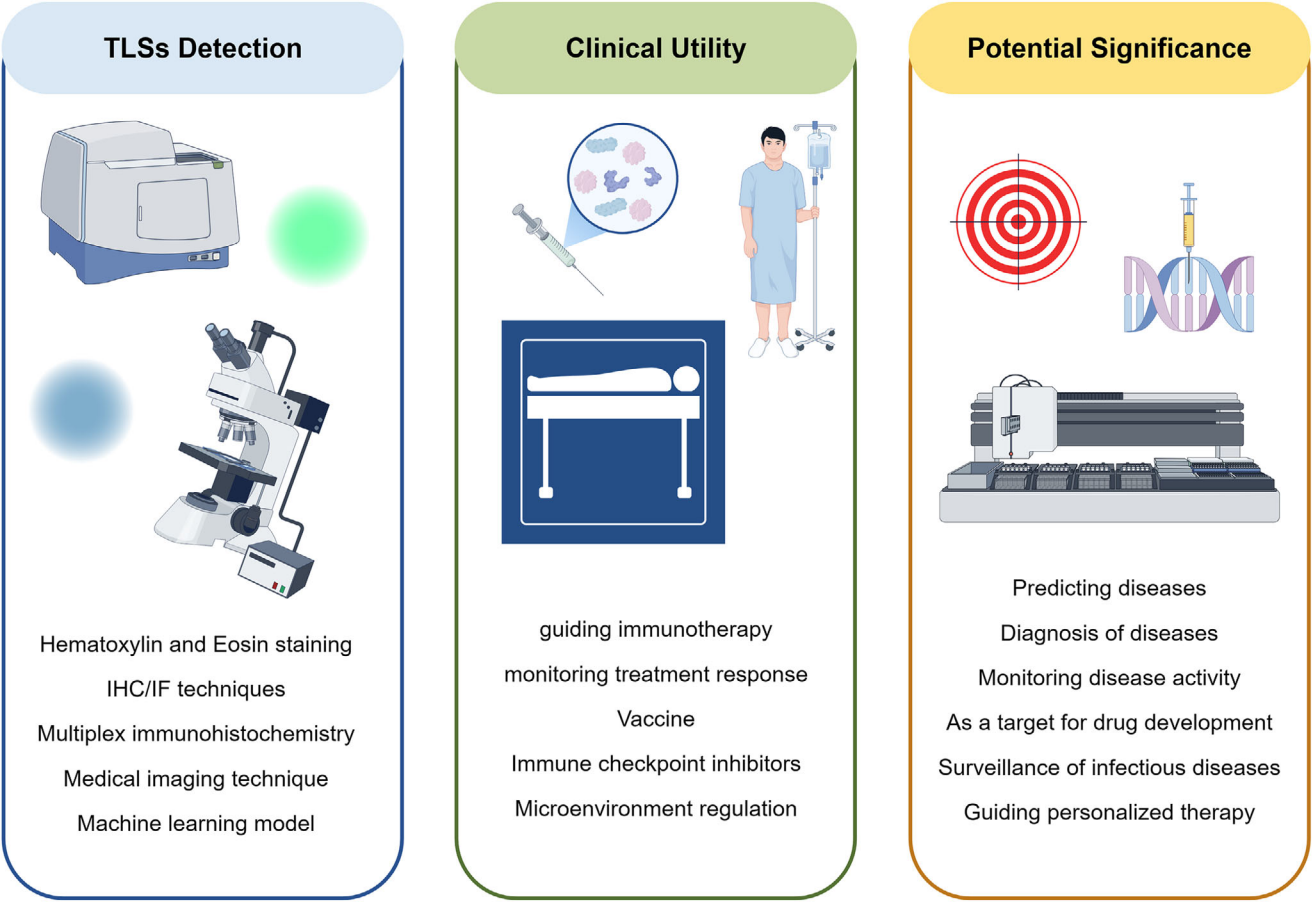
Overview of the detection approaches, clinical applications, and potential significance of TLSs. This figure summarizes three core aspects of TLS research: their clinical utility, their broad potential significance, and the main detection techniques. The left panel lists common TLS detection technologies, covering H&E staining, IHC/IF, multiplex immunohistochemistry, medical imaging, and machine learning models. The central panel outlines translational clinical utility, including immunotherapy guidance, treatment response monitoring, vaccine and checkpoint inhibitor development, and tumor microenvironment remodeling. The right panel describes broad research value of TLS for disease prediction, diagnosis, activity monitoring, targeted drug development, infectious disease surveillance, and personalized therapy design. This image was created by Figdraw.

TLSs demonstrate significant associations with disease progression and clinical outcomes. Given their promising potential as prognostic biomarkers, the development of robust detection methodologies becomes imperative, yet no single technique currently addresses all scientific and clinical needs, as each method balances trade‐offs in invasiveness, resolution, and scalability. This review critically examines pivotal technological advances in TLSs detection, first synthesizing core features of each approach in a comparative framework (Table 2) to guide method selection, then delving into the mechanistic and translational details of key techniques, with the aim of providing actionable insights for both scientific investigation and clinical translation (Figure 2).

**TABLE 2:** Comparison of detection methodologies for TLSs.

Method	Key principles/Markers	Advantages	Limitations	Typical applications	Refs.
Hematoxylin and Eosin (H&E) staining	Histological visualization of tissue architecture; identifies lymphoid aggregates, germinal center‐like structures.	Simple operation, low cost; Wide availability in clinical laboratories; Enables preliminary assessment of TLS maturity.	Limited to 1–2 targets; no cellular phenotypic information; Only rough quantification of TLSs; prone to oversights in whole‐slide analysis.	Initial screening of TLSs in routine histopathological samples (e.g., RA synovium, SLE kidney); Large‐scale epidemiological studies of TLS prevalence.	[8, 75, 77, 78, 79, 80]
Immunohistochemistry (IHC)/Immunofluorescence (IF)	Labels 3–4 specific biomarkers (e.g., CD3/CD20 for T/B cells; CD21 for FDCs; CXCL13 for chemokine signaling).	Provides cellular phenotypic and spatial information of TLS components; Higher accuracy in TLS identification vs. H & E; Enables analysis of TLS functional states.	Dependent on high‐quality antibodies (risk of nonspecific staining); Complex protocol requiring stringent experimental conditions; Fixed tissue samples fail to capture in vivo TLS dynamics.	Characterization of TLS cellular composition in autoimmune diseases (e.g., SS salivary glands, MS meninges); Correlation of TLSs with disease staging/prognosis in cancer (e.g., colorectal cancer tumor tissues).	[53, 80, 81, 82]
Multiplex Immunohistochemistry (mIHC)	Labels ≥5 biomarkers (e.g., CD4/CD8/CD20/PD‐1/CD21) via techniques like Opal‐TSA; enables spatial co‐localization analysis.	High‐dimensional profiling of TLS immune cell interactions; Standardized protocols (e.g., Opal‐TSA for 7 markers); Predicts therapeutic response (e.g., PD‐L1 expression in TLSs and immunotherapy efficacy).	Requires specialized equipment and trained personnel; Risk of tyramide signal over‐reaction (TSA‐based methods); Fluorophore limitations restrict marker diversity.	Deep phenotypic analysis of TLSs in cancer; Investigation of TLS microenvironment heterogeneity in autoimmune diseases.	[53, 77, 83, 84, 85, 86, 87]
Medical imaging techniques	Non‐invasive visualization; PET uses radiolabeled antibodies (e.g., Zr‐89‐anti‐CD3) to detect TLS‐associated immune cells.	Non‐invasive, real‐time monitoring of TLSs in vivo; CT offers high spatial resolution for TLS localization; Potential for whole‐body TLS screening.	Low accuracy/specificity for direct TLS detection; High cost; limited availability of radiolabeled probes; Fails to distinguish mature vs. immature TLSs.	Localization of acute TLSs (e.g., inflammatory foci in autoimmune diseases); Assessment of TLS‐associated immune infiltration in tumors (e.g., PET detection of CD3+ T cells in cancer).	[53, 88, 89, 90, 91]
Machine learning models	Automated analysis of H&E/spatial transcriptome data; algorithms (e.g., HookNet, support vector classifiers) detect/count/classify TLSs.	High accuracy; Reduces inter‐observer variability; objective quantification; Applicable to routine H&E slides.	Dependent on large, high‐quality training datasets; Limited generalization across tissue types; Requires computational expertise for model development.	Large‐scale TLS quantification in cancer cohorts; TLS‐based prognostic stratification.	[79, 89, 92, 93]

### 3.1 Hematoxylin and Eosin Staining

H&E staining, or hematoxylin and eosin staining, is one of the most commonly used staining methods in histology and pathology. H&E staining techniques typically can mark 1–2 targets [77]. H&E staining can identify typical TLSs structures, including distinct T cell zones (CD3^+^ T cell clusters) and B cell zones (CD20^+^ B cell clusters forming follicles), observe the presence of GC‐like structures, and assess the maturity of TLSs [8, 75, 78]. At present, H&E staining is widely used for TLSs detection due to its simplicity and cost‐effectiveness. However, it offers limited information, has certain subjective variability, and only allows for rough quantification [79, 80]. Moreover, evaluating TLSs on H&E sections is laborious, requiring pathologists with extensive morphological expertise for diagnosis, and counting TLSs across whole slides is prone to oversights.

### 3.2 Immunohistochemistry (IHC)/Immunofluorescence (IF) Techniques

Immunohistochemistry/Immunofluorescence (IHC/IF) techniques can label 3–4 markers on tissue sections, providing richer cellular phenotypic information than traditional H&E staining. This aids in identifying and locating TLS constituent cells, such as CD20^+^ GCs, PDPN^+^ FAP^+^ DAPI^+^ fibroblast networks, and CD3^+^ CD26L^+^ T cells, among others [80]. IHC/IF technology enables the simultaneous detection of multiple cellular markers, allowing for more accurate identification and characterization of the cellular composition and spatial architecture of TLSs. For instance, by detecting cellular markers and chemokines such as CD3, CD19, CXCL13, and CXCL21, researchers can gain deeper insights into the developmental stages and functional states of TLS [53, 81]. In addition, IHC/IF techniques were used to analyze TLSs characteristics in tumor tissues and adjacent normal tissues from colorectal cancer (CRC) patients. The results showed that the number and maturity of TLSs in tumor tissues were significantly higher than in normal tissues, and were closely related to tumor staging and patient prognosis. By detecting PD‐L1 expression levels in TLSs, the response of patients to immunotherapy could be predicted [82]. However, IHC/IF techniques also have limitations, such as the need for high‐quality antibodies to ensure specificity and sensitivity in detection, and the complexity of the procedures, which require stringent experimental conditions and skilled personnel. Moreover, since IHC/IF is typically performed on fixed and sectioned tissue samples, it may not fully capture the dynamic changes and functional status of TLSs in vivo [80].

### 3.3 Multiplex Immunohistochemistry (mIHC)

To overcome the limitations of IHC/IF, multiplex immunohistochemistry (mIHC) technology enables the labeling of more than five biomarkers [53], such as CD3, CD8, CD20, PD‐1, facilitating the identification of different immune cell types and their distribution and interactions within TLSs [77, 83]. For instance, by detecting the FDC marker CD21 and the cytotoxic T cell marker CD8, co‐localization analysis of subpopulations within TLS can be conducted, providing deeper insights into the cellular composition and function of TLS [83]. The OpalTM‐TSA mIHC system provides a standardized experimental protocol for the detection of TLSs characterization, capable of simultaneously marking 7 markers including CD4, CD8, CD19, CD21, DC‐LAMP, PNAd, and DAPI [84]. In a study, the correlation between PD‐L1 expression in non‐small cell lung cancer (NSCLC) tissues and TLSs was detected by mIHC, finding that tumors with high PD‐L1 expression had higher density and maturity of surrounding TLSs, and were closely related to immune‐related adverse reactions and treatment response in patients [85]. This indicates that mIHC technology has potential value in predicting tumor immune treatment response and evaluating patient prognosis. However, mIHC experiments involve the optimization of multiple antibody combinations, detection of fluorescent signals, and image analysis, requiring specialized personnel and equipment to ensure experimental success and result reliability [86]. While methods like tyramide signal amplification (TSA) can enhance signals, they may also cause tyramide signal over‐reactions, and limitations in fluorophores can affect the diversity and intensity of labeling, impacting detection sensitivity and specificity [87].

### 3.4 Medical Imaging Technique

While H&E staining, IF, IHC, and mIHC are invasive methods for detecting TLSs, medical imaging techniques such as computed tomography (CT), positron emission tomography (PET), single‐photon emission computed tomography (SPECT), and magnetic resonance imaging (MRI) offer non‐invasive and real‐time alternatives [53]. To overcome the limitations of conventional methods, researchers are exploring novel medical imaging techniques. For instance, the TLS‐PAT AI tool developed by Le Rochais et al. classifies TLSs maturation stages through dual IHC staining (CD21/CD23), offering higher specificity and sensitivity compared to traditional H&E staining, thus enabling more accurate identification of TLS maturation stages [88]. In addition, multiresolution convolutional neural networks based on deep learning (e.g., HookNet) can automatically identify mTLSs, providing a more efficient and accurate means for pathological detection [89]. CT scans are fast and can complete whole‐body scans in a short time. They have high spatial resolution in detecting TLSs, allowing for clear visualization of the details of structures such as bones and soft tissues, which is of great significance for the rapid diagnosis and localization of acute TLSs [90]. Due to limitations in accuracy and specificity, few imaging applications are used for direct TLS detection. Researchers have developed materials for imaging single cells in TLSs. For example, Zr‐89‐labeled deferoxamine‐modified anti‐CD3 mAb detected CD3^+^ T cell infiltration in tumors via PET [91]. However, it is argued that imaging of individual lymphocytes does not effectively indicate the presence of TLSs [53].

### 3.5 Machine Learning Model

Zhe Li et al. developed and validated a machine learning model for automatic detection, counting, and classification of TLSs from H&E‐stained histopathological images. The model achieved a diagnostic accuracy greater than 95% in 1924 patients with gastrointestinal cancer, and quantitative TLSs scoring was an independent prognostic factor related to survival [79]. Conventional TLSs assessment methods are limited by inter‐observer variability and the high complexity and cost associated with specialized imaging techniques [79]. In contrast, machine learning models can be directly applied to routinely stained H&E images without the need for additional specialized imaging techniques, significantly reducing costs and operational complexity. In addition, manual TLSs assessment is subjective, whereas machine learning models provide more objective and consistent detection and classification results. For example, one study measured inter‐observer variation in visually interpreted H&E‐stained manual TLSs and GC annotations to investigate subjectivity in human TLSs detection and evaluate model performance in that context [89]. Zhang Xiaonan and colleagues' research analyzed data from 13 independent breast cancer cohorts, using single‐cell RNA sequencing and machine learning algorithms to identify key TLS‐related genes and develop a TLS‐based predictive model. The model is capable of dividing patients into high‐risk and low‐risk groups and outperforms conventional models in predicting overall survival (OS) [92]. These findings demonstrate that machine learning models offer high accuracy, objectivity, and operationality in TLSs detection.

### 3.6 Other

Recent studies have utilized spatial transcriptome data to construct a support vector classifier model for TLSs prediction, finding that most TLSs biomarkers are immunoglobulin genes, confirming their significance in TLSs identification [93]. Single‐cell‐RNA‐sequencing (scRNA‐seq) and spatial‐enhanced‐resolution‐omics‐sequencing (Stereo‐seq) showed that TLS+ tumors are enriched with IgG plasma cells, while TLS‐ tumors have IgA plasma cells [94]. Another scRNA‐seq study revealed that diverse TLS conditions in TME lead to distinct intercellular communication and metabolic heterogeneity, with survival variations [95]. In HNSCC, single‐cell RNA, antigen receptor sequencing, and spatial transcriptomics showed that mTLSs contain stem‐like T cells and B cells at various stages [96]. Single‐cell RNA sequencing and spatial transcriptomics have identified a subset of IGLL5 B cells in bladder cancer (BCa) that can disrupt the integrity of TLS and weaken immunotherapy responses [97]. High‐resolution techniques including TLS‐specific signatures, spatial transcriptomics, and scRNA‐seq identified eighteen cell types in TLS‐associated regions of breast cancer, and gene expression differences were calculated by comparing TLS+ versus TLS‐ areas [98]. Clinically, high IRF4 expression predicts worse outcomes, linked to immature TLS and fewer CD8^+^ T cells infiltration [99]. Mechanistically, scRNA‐seq analyses revealed that IRF4 signaling is active in immature B cells and opposes TLS maturation [99]. Spatial transcriptomics further confirmed IRF4 enrichment within TLS regions, notably separated from HEVs and mTLS compartments [99]. Finally, integration of RNA and single‐cell sequencing data yielded a TLSscore model, whose gene signature matched the spatial distribution of TLS and proved capable of predicting survival and immunotherapy response in HNSCC patients [100].

## 4 Mechanisms Underlying TLSs Formation in Autoimmune Pathogenesis and Cancers

TLSs arise as organized lymphoid aggregates in non‐lymphoid tissues under chronic inflammatory conditions, driven by sustained chemokine/cytokine signaling and dysregulated immune activation [101, 102, 103]. These ectopic formations frequently develop in autoimmune disorders, where persistent inflammation may infiltrate and foster GC‐like architectures. The formation of TLSs is not a random event but a coordinated, multi‐stage process guided by specific molecular pathways and immune cell interactions, which can be divided into three key stages: stromal cell activation, immune cell recruitment, and TLSs maturation [104].

### 4.1 Stromal Cell Activation

Stromal cells, primarily fibroblasts and endothelial cells (ECs), act as the foundational organizers of TLSs. Their activation into LTo‐like cells is the critical first step, establishing a supportive microenvironment for subsequent immune cell aggregation and organization [105, 106].

The activation of these “immunofibroblasts” is driven by a network of cytokines and cell‐contact signals. Key cytokines include IL‐13 (signaling through IL‐4R), IL‐17, and IL‐22, which promote fibroblast proliferation and specialization [52, 60, 107]. A key initiating signal comes from the lymphotoxin (LT) pathway. Fibroblasts specifically bind to LTα1β2 and LIGHT by LTβR, stimulating their proliferation [103]. LTα3 also binds to LTβR and mediates TLSs development, which is essential for TLSs development [108], as LTβR deficiency severely blocks TLSs formation [109].

A central consequence of stromal activation across different tissues is the stable production of homeostatic chemokines. Activated stromal cells, functioning as LTo, secrete key chemokines such as CXCL13, CCL19, CCL21, and CXCL12 [103, 106]. The transcription of CXCL13, CCL19, and CCL21 mRNA was significantly upregulated before TLSs development and peaked when TLSs were fully developed [110], establishing the essential chemotactic gradient for recruitment. The chemokines such as CXCL12, CXCL13, CCL19, and CCL21, bind to receptors CXCR5 and CCR7 on lymphocytes, orchestrating the coordinated recruitment of CD3^+^ T cells and B220^+^ B cells to the newly forming TLSs site [103, 106]. The critical role of this axis is confirmed by studies showing that CXCL13 inhibitors depress TLSs formation [109].

In different organs, the stromal cells that trigger TLSs formation appear tissue‐specific. In lupus nephritis, nucleosome‐containing immune complexes in the mesangial matrix activates mesangial cells, inducing a broad chemokine profile (CCL2, CCL7, CCL20, CXCL1, CXCL2, CXCL5) that initiates a cascade of neutrophil, macrophage, and lymphocyte infiltration into the renal stroma [111]. In mice with melanoma, cancer‐associated fibroblasts express high levels of CXCL12 and CXCL13, leading to the aggregation of T cells and B cells in the PDPN^+^ network and inducing follicle formation in TLSs [106]. And CXCL13 inhibitors depress TLSs formation [109]. Notably, IL‐17‐induced CXCL12 can recruit B cells and induce follicle formation even in the absence of CXCL13^+^ FDCs [112].

During the activation of local fibroblasts, these cells in the tissue acquire lymphoid matrix characteristics, a process that necessitates the involvement of chemokines [101]. Researchers have demonstrated that LTi cells have been shown to express lymphotoxin, which activates the lymphotoxin receptor on fibroblast stromal cells, resulting in the induction of chemokines CCL19, CCL21, and CXCL13 [113]. These chemokines subsequently bind to their respective receptors, facilitating fibroblast activation and the development of lymphoid matrix characteristics, thereby attracting more lymphocytes to support TLSs formation [114]. Consequently, fibroblast activation is crucial for the creation of TLSs [115, 116].

In summary, the activation of stromal cells into an LTo phenotype is the foundational trigger for TLS neogenesis. This process is universally driven by core signaling pathways, especially the LT‐LTβR axis and a network of polarizing cytokines (e.g., IL‐13, IL‐17), which work together to reprogram stromal cells. The indispensable output of this reprogramming is the sustained production of key lymphoid chemokines such as CXCL13, CCL19, and CCL21, which establish the initial chemotactic blueprint for lymphoid organization. This successfully established chemokine field sets the stage for the next critical phase: the coordinated recruitment and subsequent activation of diverse immune cells, which will amplify and mature the initial structure into a functional TLS.

### 4.2 Immune Cells Recruitment and Amplification

Following stromal cell activation, the recruitment of immune cells marks the transition of TLSs development into a self‐sustaining phase. This process is closely associated with the developmental maturity of TLSs [28, 117]. The formation of TLSs relies on the coordinated action of chemokines, which can be produced by both stromal and hematopoietic cells to establish the organizational hubs of TLSs [104].

A key feature of this stage is the establishment of a positive feedback loop. While stromal‐derived chemokines initiate recruitment, the infiltrating immune cells themselves become powerful sources of lymphoid chemokines, thereby amplifying the TLS formation signal. Notably, these macrophages specifically localize within TLSs, suggesting that the TLSs microenvironment may recruit and regulate such hematopoietic cells via specific mechanisms. Further studies have revealed that hematopoietic cells within TLSs, such as CD8^+^ T cells, also secrete chemokines like CXCL13, directly contributing to TLSs maturation [118, 119, 120, 121, 122]. These findings indicate that chemokines derived from stromal cells may be insufficient to ensure the initial formation of TLSs, and that coordinated contributions from multiple hematopoietic cell populations are likely a universal feature of TLSs development, but their specific cellular composition may vary depending on the type of inflammatory stimulus [104].

The recruitment and function of specific immune cells are critical for shaping the TLSs. DCs and macrophages are not only recruited but are essential for sustaining the TLSs microenvironment through antigen presentation and cytokine production. For instance, in a murine model of *Salmonella enterica* infection, CXCL13^+^CX3CR1^+^ tissue‐resident macrophages promoted TLSs formation and the initiation of local GC responses by effectively recruiting and activating B and T cells at the infection site through antigen presentation [118]. Furthermore, B cells are actively recruited via CXCL13‐CXCR5 signaling and further contribute to TLS stability by serving as APCs and providing LTα1β2 signals back to stromal cells [73, 123, 124]. In addition, the recruitment of CD4^+^ T cells, particularly T follicular helper (Tfh) cells, is pivotal. In patients with lupus nephritis, the presence of follicular T cell‐like CD4‐T cells in kidney biopsy samples is linked to a lower estimated glomerular filtration rate [125], implying a potential involvement of TLSs in the disease [30].

Furthermore, research on lung adenocarcinoma and the chemokine CXCL13 suggests that TLSs formation is initiated by CXCL13‐mediated recruitment of lymphoid tissue, which induces HEVs formation [20], immune cells recruit, essential chemokines production, and ultimately facilitates the further maturation of TLSs [104]. The observations imply that circulating cells or humoral factors might play a role in the development of TLSs in autoimmune disorders.

### 4.3 TLSs Maturation

In chronic inflammatory environments, TLSs progression and maturation correlate with the severity of disease and the extent of kidney damage [126]. This correlation has been shown in kidney injury in mice [127], as well as in patients with lupus nephritis, IgA nephropathy, and transplanted kidneys [128]. Contrastingly, the presence and maturation of TLSs in many cancer types, including renal cell carcinoma, correlate with better survival and response to treatment [28]. TLSs maturation is a complex process that transforms a loose lymphoid aggregate into a highly organized, functional structure, culminating in the formation of specialized HEVs [129]. HEVs are a critical hallmark of TLSs maturity, acting as a gateway for the continuous recruitment of naive T and B lymphocytes from the bloodstream [129]. As TLSs matures, the formation of HEVs further enhances immune cell recruitment, which in turn supports the ongoing maturation and functional diversification of the TLSs [106]. Collectively, TLS maturation is mainly regulated by specialized stromal cells, immune cell subsets, and their coordinated crosstalk within local inflammatory microenvironments. Key molecular signals including homeostatic chemokines and inflammatory cytokines jointly drive the progressive organization and functional maturation of TLSs.

In addition, the development of TLSs is believed to arise in response to chronic inflammatory signals in a manner dependent on antigens, with the presence of antigens being apparent in certain autoimmune diseases [29]. TLSs presence in target organs—such as the kidney in lupus nephritis, the synovium in RA, and the central nervous system (CNS) in SLE and MS—highlights their role in sustaining local antigen‐specific immune responses [130, 131, 132]. This antigen dependence is further corroborated by observations that TLSs typically resolve following antigen clearance [104, 107, 133]. However, the mechanisms governing potential antigen‐independent induction, or the long‐term maintenance of TLSs in established disease, remain a significant knowledge gap. Additional studies are needed to shed light on the role of antigen‐independent TLSs induction, to assess if these TLSs are different from those induced by antigen‐specific lymphocytes, and to delve into the mechanisms behind their induction and maintenance of TLSs when antigens are absent.

In summary, the maturation of TLSs depends on the development of HEVs, while its persistence relies on the availability of antigen. The widespread observation of TLSs across diverse autoimmune disorders implies a common pathogenic mechanism driven by chronic antigen exposure [130, 131, 132]. Future research must clarify the checkpoints among TLSs maturation, maintenance, and resolution to identify new therapeutic targets.

The specific mechanisms that lead to the formation of TLSs are still under investigation, but they are known to be related to a localized, dysregulated immune‐inflammatory response. There are only a handful of studies that have examined the factors leading to TLSs formation in autoimmune diseases and cancers, with most studies merely characterizing TLSs and correlating their existence and/or maturity level to disease outcomes. Consequently, there is a considerable lack of knowledge regarding the formation processes and mechanisms of TLSs in autoimmune diseases and cancers, as well as the clinical factors that may influence TLSs dynamics.

## 5 Role of TLSs in Autoimmune Diseases and Cancers

**FIGURE 3:**
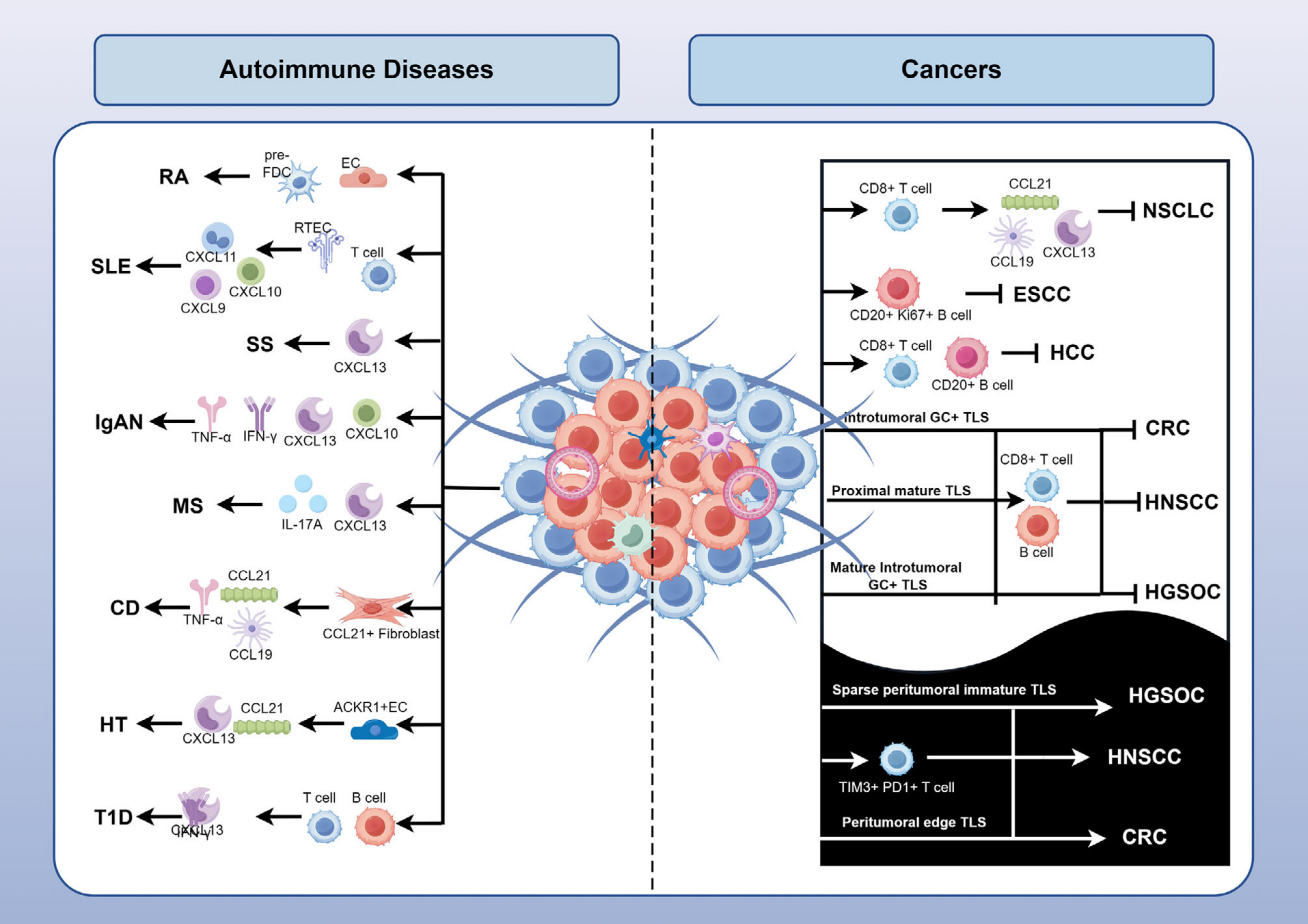
Pathogenic and dual roles of TLSs in autoimmune diseases and cancers. This figure summarizes the key mediators (cytokines, chemokines, cell types) underlying TLS function in representative autoimmune diseases (left, pathogenic role) and cancers (right, dual role). The left branch depicts cellular sources and inflammatory mediators that trigger autoreactive lymphocyte activation and tissue damage across systemic and organ‐specific autoimmune disorders (RA, SLE, SS, IgAN, MS, CD, HT, T1D). The right cancer branch distinguishes two functionally distinct TLS subsets. Intratumoral mature TLS with germinal centers recruit functional CD8^+^ T and CD20^+^ B cells to suppress tumor progression in NSCLC, ESCC, and HCC. Peritumoral immature edge TLS accumulate TIM3^+^PD1^+^ exhausted CD8^+^ T cells, driving immune escape and tumor invasion in CRC, HGSOC, and HNSCC. This image was created by Figdraw.

TLSs play a diverse and often contrasting roles across autoimmune diseases and cancers, largely defined by their unique cellular and molecular microenvironment. Figure 3 provides a visual framework of the key cell types and cytokines that shape TLS formation and function within these distinct pathological contexts, serving as a visual guide for the detailed discussions that follow.

### 5.1 Relationship Between TLSs and Autoimmune Diseases

The mechanistic interplay between TLSs and autoimmune pathogenesis remains incompletely defined, particularly regarding conserved developmental pathways across diverse tissues and disease states. Clinically, TLSs exhibit context‐dependent roles contingent on disease‐specific pathophysiology. TLSs have been identified in nearly all organ‐specific autoimmune disorders, including RA [134], LN [135], TID [136], Crohn's disease (CD) [137], and Sjögren's syndrome (SS) [138]. Across clinical studies, TLSs presence consistently correlates with elevated disease activity and severity [71], notably in LN and RA where TLSs harbor autoreactive B cells capable of pathogenic autoantibody production [139, 140, 141]. Concurrently, TLS‐associated autoreactive T cells drive tissue damage through dysregulated cytokine release [29]. This section systematically examines the clinical and immunological associations of TLSs in prototypical autoimmune conditions, including SLE, IgA nephropathy, RA, SS, MS, CD, Type 1 Diabetes (T1D), and Hashimoto's Thyroiditis (HT) (Table 3).

**TABLE 3:** Summary of TLS phenotypes, regulatory mediators and context‐dependent functions in autoimmune diseases and cancers.

Category	Disease	Defining TLS characteristics & cellular composition	Molecular regulators of TLS formation and function	Functional roles	Disease‐specific functional consequences and clinical significance	Therapeutic relevance	Refs.
Systemic autoimmune diseases	SLE	Renal (lupus nephritis) TLS; FDC networks with CD4+ T‐ and CD20+ B‐cell aggregates	CXCL13–CXCR5; LTα1β2–LTβR	Local autoantigen presentation niche driving autoreactive B/T‐cell activation (pathogenic)	TLS abundance tracks with severe lupus nephritis and irreversible renal damage	CXCL13/CXCR5/LTβR blockade suppresses TLS neogenesis	[31, 140, 143, 144, 145]
	RA	Synovial TLS with germinal centers; segregated B/T zones, AID+ affinity‐matured B cells	TNF‐α (↑CCL21); JAK–STAT	Sustains autoantigen presentation and local affinity maturation (pathogenic)	Predicts chronic synovitis, flares and worse prognosis	Anti‐TNF and JAK inhibitors reduce TLS burden	[29, 123, 146, 147, 148, 149]
	SS	Salivary‐gland TLS with complete GC and FDC networks	BAFF/APRIL; TLR7/9	Drives local autoantibody production and glandular destruction (pathogenic)	Progressive, irreversible exocrine gland dysfunction	BAFF inhibitors and TLR antagonists disrupt TLS	[140, 150, 151]
Organ‐specific autoimmune diseases	IgAN	Renal interstitial TLS; CD8+/CD20+ lymphocyte aggregates	NF‐κB; CXCL12–CXCR4	Amplifies interstitial inflammation and sustains autoreactive pools (pathogenic)	TLS density correlates with fibrosis and renal progression	NF‐κB or CXCR4 inhibition limits TLS‐driven injury	[140, 152, 153]
	MS	Meningeal/perivascular TLS in CNS lesions; B cells and macrophages	CCL2–CCR2; IFN‐γ	Recruits peripheral autoreactive T cells into CNS niches (pathogenic)	Sustained demyelination and progressive neurological decline	CCR2 targeting/IFN‐β modulate CNS TLS	[154]
	Crohn's Disease	Mesenteric TLS adjacent to inflamed intestinal segments	TNF‐α; adipokine‐derived chemokines	Drives chronic intestinal inflammation and impairs lymphatic drainage (pathogenic)	Aggravates fibrosis and recurrent mucosal ulceration	Anti‐TNF therapy reduces mesenteric TLS	[155, 156, 157, 158]
	T1D	Rare islet‐resident TLS in humans; functional in NOD mice	Islet chemokine cascades (not fully defined in human)	Accelerates β‐cell autoimmunity (context‐dependent pathogenic)	Linked to accelerated insulin loss; human relevance limited by low prevalence	TLS‐targeted effect unclear given low human prevalence	[123, 159, 160, 161, 162, 163]
	Hashimoto's thyroiditis	Thyroid TLS organized by ACKR1+ EC and CCL21^+^ fibroblasts	CCL21–CCR7; stromal organizer signals	Recruits autoreactive T cells and sustains glandular injury (pathogenic)	Progressive follicle destruction leading to hypothyroidism	CCL21 neutralization blocks TLS assembly	[164, 165, 166, 167, 168]
Cancer	NSCLC	Mature intratumoral TLS with complete GC architecture	CXCL13; CCL19/CCL21; LTα1β2–LTβR	Coordinates anti‐tumor adaptive immunity (protective)	TLS maturity correlates with prolonged OS and ICB response	Robust biomarker predicting immunotherapy benefit	[28, 33, 35, 36, 169, 170, 171]
	Colorectal Cancer	Intra‐ vs. peri‐tumoral TLS with distinct activity	CCL19/CCL21; CXCL13; CCL28–CCR10	Mature intratumoral TLS drives anti‐tumor cytotoxicity (protective, context‐dependent)	Intratumoral TLS confers longer relapse‐free survival	Prognostic marker after adjuvant chemotherapy	[94, 172, 173, 174, 175]
	Ovarian Cancer (HGSOC)	Sparse, scattered TLS within immunosuppressive microenvironment	SCD1; ATF3; CCL4	Limited anti‐tumor activity offset by dominant immunosuppression (complex)	Mostly associated with unfavorable prognosis	Inducing TLS maturation may enhance ICB	[154, 176, 177, 178, 179]
	ESCC	Minimal intratumoral TLS (mTLS); composition‐dependent impact	Immunoglobulin‐subtype transcriptional regulators; stromal chemokines	Drives intratumoral Ig production and T‐cell activation (protective)	High mTLS abundance predicts extended survival	TLS subset quantification enables patient stratification	[79, 180, 181, 182, 183, 184, 185, 186]
	HNSCC	TLS enriched with progenitor T/B cells; proximity to exhausted TILs matters	CXCL13; CCL19/CCL21	Rescues dysfunctional TILs and restores immune surveillance (protective)	TLS–tumor proximity predicts durable ICI response	Spatial distribution and maturity guide therapy selection	[187, 188]

#### 5.1.1 TLSs in SLE

SLE is a multisystem autoimmune disorder characterized by antinuclear autoantibodies and immune complex deposition, with LN representing its most severe complication [140, 188]. An analysis of SLE patients revealed that those with lupus nephritis

 exhibited different classes of disease severity based on the expression of CD20, CD3, and CD21 in the kidneys [53]. The presence of CD20 and the formation of TLSs were associated with a longer disease course, a poorer response to therapy, and higher levels of CXCL13, a chemokine linked to B cell recruitment and renal function impairment. Importantly, CXCL13 levels were found to be positively correlated with the severity of tissue inflammation and the number of B cells in the renal tissue, highlighting its potential role as a marker for disease progression and treatment response in lupus nephritis patients [144]. Moreover, TLSs have been identified in renal tissues of both SLE/LN patients and murine disease models. TLSs formed in a lupus‐prone mouse model exhibit a node‐like gene expression pattern, with FDC‐B cell interactions and MIDC‐8^+^ dendritic cells localized to the T cell region [143]. SPECT imaging detected pancreatic TLSs in these models, with subsequent immunohistochemical validation [142]. Clinically, renal TLSs presence in LN patients correlates with elevated serum CXCL13 levels, implicating FDC and FDC‐derived CXCL13 in TLSs may play a role in TLSs development [144]. These findings collectively suggest DCs may orchestrate TLSs development to potentiate localized adaptive immune activation.

Protein phosphatase 2A (PP2A) is a widely distributed serine/threonine phosphatase that participates in regulating multiple signaling pathways [189]. Due to its aberrant expression, PP2A has been implicated in various diseases [190, 191, 192, 193], inhibition of PP2A has been implicated in the treatment of various diseases. LB‐100, a small molecule inhibitor of PP2A, has demonstrated anti‐tumor therapeutic effects and high safety profile in preclinical experiments [31]. Prior studies have demonstrated an increased expression of PP2A in both T cells and B cells among individuals with SLE [194, 195]. T or B cell‐specific PP2A knockout mice exhibited reduced autoantibody production and splenomegaly in SLE models [195, 196]. These findings indicated the potential benefit of PP2A inhibition in treating the disease, but its role in LN remains unclear. Yang et al. employed the PP2A inhibitor LB‐100 to treat both MRL/Lpr mice and R848‐induced BALB/c mice, they indicated that LB‐100 administration led to reduced spleen enlargement, decreased deposition of immune complexes, ameliorated renal damage, and improved kidney function in both spontaneous and R848‐induced lupus mouse models [31]. Importantly, Yang et al. observed the formation of TLSs in the kidneys of two distinct lupus mouse models, and they demonstrated that LB‐100 prevents renal damage by inhibiting TLSs formation in two distinct lupus mouse models [31]. Renal tubular epithelial cells (RTECs) produce T cell chemokines (CXCL9, CXCL10, CXCL11), thereby promoting the infiltration of T cells and the formation of TLS in LN, but LB‐100 inhibits the production of T cell chemokines secreted by RTECs, thus mitigating renal damage [31].

#### 5.1.2 TLSs in IgA Nephropathy

IgA nephropathy is an immune‐mediated renal disorder characterized by glomerular IgA deposition and progressive kidney dysfunction. Histopathological analyses reveal TLSs in renal biopsies of IgA nephropathy patients, with prevalence correlating to disease severity [151]. These ectopic lymphoid aggregates demonstrate organized CD4^+^/CD8^+^ T cell zones and CD20^+^ B cell clusters, surrounded by DC‐SIGN^+^ dendritic cells at the periphery [140].

TFH cells are a special subpopulation of effector CD4^+^ T cells, which are most commonly recognized as CXCR5^+^CD4^+^ cells and also express inducible T‐cell co‐stimulator (ICOS) molecule and the coinhibitory molecule programmed cell death protein 1 (PD‐1) [197]. Although the orchestration of the lymphotoxin and chemokine seems to be critical for TLS formation, TFH cells are also indispensable for the formation and maintenance of GCs of TLSs [198]. TFH cells migrate to B cell follicles under the action of CXCR5 ligand chemokine CXCL13, and then regulate the differentiation of B cells through ICOS–ICOS ligand [199].

IL‐21, a canonical functional mediator of TFH cells, is often observed in chronically inflamed tissue, such as lupus nephritis, Sjogren's syndrome, and atherosclerosis [200]. IL‐21 neutralization inhibits GC maturation, reduces autoantibody production, and delays the progression of glomerulonephritis [201]. Furthermore, through interacting with differentiated fibroblasts, TFH cells are engaged in both the early inflammatory phase and late fibrotic phases by IL‐21 [202]. In addition, the expression of IL‐21 receptor (IL‐21R) is detected in infiltrating lymphocytes of human pulmonary fibrosis specimens, suggesting that IL‐21‐responsive lymphocytes might be related to the progression of pulmonary fibrosis [203]. However, the underlying mechanisms of IL‐21 and renal fibrosis remain poorly defined.

Gang Xu et al. demonstrated that TFH cells contribute to TLSs formation and renal fibrosis by IL‐21, they detected the circulating TFH cells from 57 IgA nephropathy patients and found that the frequency of TFH cells was increased in IgA nephropathy patients with renal TLSs and also increased in renal tissues from the ischemic‐reperfusion‐injury (IRI)‐induced TLSs model [152]. The ICOS is one of the surface marker molecules of TFH. Remarkably, the application of an ICOS‐neutralizing antibody effectively prevented the upregulation of TFH cells and expression of its canonical functional mediator IL‐21, and also reduced renal TLSs formation and renal fibrosis in IRI mice in vivo [152].

Clinically, TLS‐positive patients exhibit elevated serum creatinine levels, severe glomerulosclerosis, tubular atrophy/interstitial fibrosis, arteriolar hyalinosis, and marked interstitial immune infiltration [151]. Mechanistically, IL‐21 promotes TFH cell accumulation within TLSs, exacerbating renal damage [152], while IL‐17A drives TLSs neogenesis and IgA‐mediated injury [204]. Furthermore, circulating soluble CD30 levels associate with TLSs burden and IgA nephropathy progression [205]. Collectively, these findings establish TLSs density as a prognostic indicator of histological deterioration and disease acceleration in IgA nephropathy pathogenesis.

#### 5.1.3 TLSs in RA

TLSs exhibit multifaceted roles in RA, a chronic autoimmune disorder afflicting 0.5%–1% of adults worldwide [123]. RA pathogenesis is marked by synovial membrane inflammation progressing to cartilage destruction and subchondral bone erosion [145, 146, 147, 148]. Histologically, RA manifests as three distinct subtypes: lymphoid (characterized by synovial T/B cell aggregates forming TLSs, associated with poor prognosis); myeloid (dominated by macrophage infiltration); fibroid (exhibiting stromal fibrosis) [71]. In the lymphoid subtype, TLSs facilitate B cell affinity maturation, suggesting their critical role in sustaining localized autoimmune responses [29]. Mechanistically, experimental evidence links TLSs formation to NIK^+^ ECs and pre‐follicular dendritic cells (pre‐FDCs), implicating shared pathways between lymph node development and inflammatory TLSs neogenesis [206]. In addition, the presence of TLSs was associated with a more severe disease course in RA. Meningeal inflammation and TLSs could represent an important fluid or imaging marker of disease activity, whose therapeutic abrogation might be necessary to stop the most severe outcomes of disease [207]. In a separate evaluation, it was found that ECs can produce NF‐κB‐inducing kinase, a key player in non‐classical NF‐κB signaling triggered by LTβ receptor signaling, which contributes to the formation of TLSs in RA [208]. This highlights the complexity of signaling pathways involved in the progression and severity of RA, underscoring the importance of TLSs in the disease's pathology. All in all, in patients with RA, the formation of TLSs is tightly correlated with worse clinical prognoses and more severe disease manifestations. Specifically, TLS‐positive patients (particularly those with the lymphoid synovial subtype) exhibit more aggressive cartilage destruction, subchondral bone erosion, and a higher risk of treatment resistance, compared to TLS‐negative counterparts. This clinical correlation further underscores the pathogenic role of TLSs in sustaining localized autoimmune responses and driving RA progression, implying a significant role for TLSs in the pathogenesis of RA.

#### 5.1.4 TLSs in SS

SS is the second most common autoimmune disease after RA, its prevalence in adults ranges from 0.2% to 3%, with a 9:1 female‐to‐male ratio [209, 210]. This chronic disorder primarily affects exocrine glands, causing symptoms like xerostomia and xerophthalmia, and can lead to systemic complications such as lymphoma and interstitial lung disease [211]. SS, an autoimmune disorder driven by pathogenic autoantibodies targeting exocrine glands, frequently develops TLSs within affected salivary and lacrimal gland tissues [140]. The TLSs in SS are primarily produced by the interaction of epithelial cells, immune fibroblasts, and infiltrating lymphocytes, which serve as unique and local sites for B cell activation and antibody affinity maturation [212]. Furthermore, it has been reported that approximately 30%–40% of SS patients have TLSs in the tissue of the salivary gland. TLSs have also been associated with lymphoma development, although this remains a matter of debate [213, 214, 215]. In a separate evaluation focusing on the serum levels of CX3CL1 in primary Sjögren's syndrome (pSS) patients, a significant elevation compared to controls was observed, indicating an inflammatory response. Histological analysis further revealed the presence of CX3CL1 and its receptor within the salivary glands of pSS patients, specifically localized within TLSs, reinforcing the role of this chemokine in the pathogenesis of pSS [150]. GC‐positive TLSs containing FDC networks and expressing AID demonstrate ongoing class‐switch recombination and somatic hypermutation, indicating antigen‐driven B cell maturation at these sites [149]. Recent studies employ advanced methodologies including single‐cell transcriptomics and spatial profiling to deconvolute TLSs cellular dynamics, revealing evolutionary trajectories from T cell clusters to functional GCs [216]. Notably, TLS‐derived IgA autoantibodies in lacrimal glands exhibit greater clonal diversity compared to IgG counterparts [217]. Furthermore, podoplanin‐high macrophages contribute to TLSs neogenesis in SS through stromal remodeling [218]. These results indicate that TLSs play a crucial role in the pathogenesis of SS.

#### 5.1.5 TLSs in MS

MS is a chronic autoimmune disorder targeting the CNS, where the presence of meningeal TLSs has emerged as a key pathological correlate of disease progression, particularly in the secondary progressive phase (SPMS) [219, 220]. TLSs are mainly found in the meninges and subarachnoid space, where they are closely associated with cortical demyelination, accelerated neuronal loss, and greater patient disability [219, 221]. Their existence marks a structured and continuous immune response within the CNS, which makes our understanding from a model of simple peripheral attack to one of local self‐perpetuating inflammation.

Through histopathological analysis of tissue from deceased MS patients, researchers have thoroughly characterized these TLSs found in the meninges. TLSs can contain CD3^+^ T cells, CD20^+^ B cells, CD138^+^ plasma cells, and CD68^+^ macrophages [222]. A defining feature is the presence of FDCs, which produce the significant B cell chemoattractant CXCL13, thereby establishing a GC‐like microenvironment that supports B cell recruitment and local antibody affinity maturation [222, 223, 224, 225]. The clinical incidence of TLSs is difficult to ascertain; while observed in up to 54% of selected SPMS cohorts, their frequency in early MS remains unclear due to the lack of available tissue [220, 226, 227, 228]. Critically, the presence and maturation of meningeal inflammation and TLSs correlate directly with elevated levels of pro‐inflammatory cytokines (e.g., TNF, IFN‐γ) and lymphoid neogenesis chemokines/cytokines (e.g., CXCL13, CXCL10, IL‐6 and IL‐10) in the cerebrospinal fluid, linking these structures to a more severe inflammatory conditions [229, 230].

The exact cellular initiators and signaling pathways driving TLS neogenesis in the MS meninges are an active area of investigation, with insights largely derived from experimental autoimmune encephalomyelitis (EAE) models. Evidence points to a significant role for IL‐17‐producing immune cells. Th17 cells have been observed in close association with developing TLSs in both EAE and human SPMS tissue, suggesting their potential in initiating stromal changes necessary for lymphoid aggregation [207, 231, 232]. In addition, the LT signaling pathway also appears to be implicated in TLSs formation in EAE [207]. A study demonstrated an upregulation of LTβ and LTβR gene expression in the CNS at EAE onset and during subsequent relapses, however, the authors found that blocking of the LTβR resulted in a reduced inflammatory infiltrate, reduced B cell aggregation within the meninges and TLSs were not observed in those cases [233]. Further evidence that LTβR signaling supports the accumulation of B cells within TLSs were demonstrated by Heiden B., et al. These findings highlight a complex process involving multiple pathways. Chronic antigen exposure in the CNS likely first draws in and activates specific immune cells like Th17 and B cells. These cells then communicate with local tissue cells using signals such as LTα1β2. This triggers the production of key chemokines like CXCL13 and CCL19/21, which ultimately guide the formation of lymphoid structures.

Functionally, meningeal TLSs act like a powerful engine that drives inflammation in the nervous system. The production of pathogenic lymphocytes and the persistent activation of surrounding microglia and astrocytes, mediated by the release of proinflammatory cytokines and chemokines, contribute to the exacerbation of neuroinflammation and the progression of MS symptoms. Emerging evidence positions TLSs as potential biomarkers for disease activity monitoring, with therapeutic TLSs disruption demonstrating promise in reducing severe neurological outcomes [207]. However, meningeal TLSs may also contain regulatory cell populations that modulate inflammatory homeostasis, suggesting their context‐dependent roles in disease progression [234]. The factors required for TLSs formation and ongoing maintenance are complex and likely involve multiple cells signaling pathways. Investigating mechanisms of TLSs formation in human MS tissue is challenging, however if we can gain a better understanding of TLSs cellular composition, particularly during different disease phases, we may gain better insights of potential immune signals required for TLSs development and maintenance.

#### 5.1.6 TLSs in CD

CD, is one of the two most common types of IBD [1, 235], with CD distinguished by its transmural, discontinuous intestinal inflammation that most often affects the terminal ileum [235]. Originally referred to as regional ileitis, CD has seen a global rise in incidence, yet its underlying etiology and pathophysiology remain incompletely understood. Increasing evidence describes the presence of a dysfunctional lymphatic system and the development of TLSs as a unique hallmark of CD (and IBDs more broadly) [154]—features that set CD apart from other autoimmune diseases where TLSs typically localize to parenchymal tissue rather than lymphatic‐associated regions.

In CD, TLSs are not randomly distributed but exhibit a highly specific localization: they form primarily in the mesentery next to inflamed ileal segments, specifically within the main afferent collecting lymphatic vessels (CLVs) and often at the valves of these vessels [155, 156]. This distinct localization is linked to continuous TNF‐α signaling, which is a main driver of inflammation in CD [156]. TNF‐α downregulates expression of lymphatic valve maintenance genes (e.g., GATA2, CX37), impairing lymphatic drainage, causing lymph leakage into the intestinal interstitium [156], and disrupting gradients of chemokines (CCL19, CCL21) that normally guide immune cell trafficking. The leaked lymph traps CCR7^+^ T cells and CXCR5^+^ B cells, initiating the aggregation of immune cells into immature lymphoid clusters that progressively mature into TLSs with typical features [156]. A study by Czepielewski et al. confirmed this mechanism in a mouse model of CD‐like ileitis, showing that TNF‐driven valve damage and TLS formation directly disrupt lymphatic transport—an impairment thought to exacerbate CD pathology by preventing immune cells from exiting the inflamed tissue [156]. Moreover, these TLSs associated with mesenteric CLV drive additional tissue dysfunction by promoting new lymph vessel growth to support their own expansion, blocking the outflow of immune cell and molecules from the ileum to draining lymph nodes, and acting as primary sites for lymph leakage and backflow into the intestinal muscularis [156].

Beyond mesenteric CLVs, TLSs in CD are also found in the mesenteric creeping fat, a specialized fatty tissue surrounding inflamed gut areas in CD patients [157]. A recent comprehensive analysis of these fat‐associated TLSs revealed that their localization is driven by adipocytes in the vicinity of TLSs, which secrete high levels of chemokines (CXCL13, CXCL16, CCL19, CCL20, CCL21) that are required to recruit immune cells for TLS formation [157].

A set of in vitro experiments further showed a synergistic effect between LPS and TNF. Specifically, their combination induces the expression of the full range of chemokines required to recruit the various cell populations involved in the formation of TLSs [157]. Although further research is needed, TLS has shown negative effects in CD. Their presence was associated with a more severe CD phenotype of the disease, whereas surgical removal of the mesentery during ileocolic resection reduced postoperative recurrence [157]. At present, the effective role of TLSs in CD onset and development remains unknown in humans. Therefore, a key goal for advancing our understanding of intestinal immunity is to better define how TLSs contribute to the disease process in CD.

#### 5.1.7 TLSs in T1D

Apart from their known roles in SLE, RA, and MS, TLSs also contribute to the pathogenesis of several other autoimmune disorders, These TLSs exhibit tissue‐specific structural forms and functions that are closely linked to disease progression.

T1D is considered to be caused by an autoimmune T cell–mediated destruction of insulin‐producing beta cells in the pancreas leading to loss of glucose homeostasis [1]. Most mechanistic insights into β cell destruction have been derived from animal models, with the non‐obese diabetic (NOD) mouse serving as the primary tool. Several important differences, however, exist between the NOD mouse model and patients, including the frequent presence of islet‐associated TLSs in the mouse model [158]. In animal models of T1D, especially the NOD mouse model, lymphoid structures in insulitis lesions are frequently seen [159]; these structures were shown to have the characteristics of fully functional TLSs, capable of supporting in situ autoreactive B lymphocyte differentiation and are suggested to be critical for promoting autoimmunity and chronic inflammation [160]. However, the absence of TLSs in patients with T1D has previously been used as an argument for a different pathogenesis of the human disease when compared with the rodent model [161]. TLSs are thought to be important for promoting antigen‐specific humoral responses during chronic inflammation in several human autoimmune conditions but have not yet been reported in patients with T1D [161]. In NOD mice, TLSs have been found to promote autoimmunity and chronic inflammation during diabetes progression [123, 162]. A case report describes the presence of insulitis and TLS‐like structures in a 66‐year‐old female patient with long term T1D [158]. A present case shows that lymphoid structures with structural characteristics of TLSs, in particular adjacent T and B lymphocyte zones, the presence of HEVs in the T cell area, and the presence of FDCs, can occur in patients with T1D, though at low frequency [158].

The role of TLSs in T1D highlights a critical gap between preclinical models and human disease: while NOD mice commonly exhibit islet‐associated TLSs that drive autoimmunity and β cell destruction, TLSs in human T1D are rare and only recently confirmed. This difference underscores the need for caution when translating TLS‐mediated mechanisms from NOD mice to patients, as the low frequency of TLSs in human pancreatic tissue raises questions about their universal contribution to human T1D pathogenesis. However, the fact that TLSs are found in some patients—even at low levels—suggests they may play a context‐dependent role, potentially contributing to disease chronicity or refractory β cell loss in specific subsets. Future research should concentrate on clarifying the incidence and functional significance of TLSs in human pancreatic samples including those from early‐stage T1D patients, and on identifying factors driving TLSs formation in human islets and NOD mice. Resolving these questions will be essential to determining whether TLS‐targeted therapies, which show promise in other autoimmune diseases, could be beneficial in T1D.

#### 5.1.8 TLSs in HT

HT is the most common autoimmune disease of the thyroid gland, defined by progressive lymphocytic infiltration of thyroid tissue, the presence of autoantibodies against thyroid peroxidase (TPO) and/or thyroglobulin (TG), and chronic thyrocyte destruction that ultimately leads to hypothyroidism [163, 236]. A typical pathological feature of HT is the presence of lymphocytic infiltrates, and even TLSs, in thyroid tissues [163].

Single‐cell RNA‐sequencing studies of HT thyroid tissue have identified three stromal cell subsets that shape the local microenvironment and drive TLS formation: ACKR1^+^ ECs, CCL21^+^ myofibroblasts, and CCL21^+^ fibroblasts [163]. Each subset occupies different histological locations and contributes to TLSs development, they might facilitate lymphocyte trafficking from the blood to thyroid tissues, and most of the CCL21^+^ cells were distributed in T cell zones of TLSs in thyroid tissues of HT patients. This localization is functionally significant, as CCL21—a chemokine critical for lymphoid organogenesis—has long been linked to SLO development: FRCs, the stromal organizers of SLOs GCs, abundantly express CCL21 and CCL19 to recruit and organize T cells [164, 165, 166]. Moreover, the studies further confirm abnormal overexpression of CCL21 in pancreas and thyroid tissues of transgenic mice can induce the formation of TLSs in the pancreas and thyroid glands [165, 167]. In patients with HT, CCL21^+^ fibroblasts localized within the TC zones of TLSs may act like LTo cells, contributing to lymphocyte recruitment and the formation of TLSs in the thyroid gland [163].

Notably, critical questions about CCL21's role in HT TLSs remain unresolved. For instance, it is unclear whether CCL21 expression by myofibroblasts or fibroblasts alone is sufficient to induce TLSs formation in the thyroid, the exact cell types and locations of CCL19 or CCL21 expressing cells in TLSs are not clear. Although CCL21 has been identified as a mediator of angiogenesis in RA [237], its potential role in promoting HEV formation in HT TLSs has not been studied. In addition, a study shows that specific stromal cells such as ACKR1^+^ ECs in HEVs, CCL21^+^ myofibroblasts, and fibroblasts appear in the thyroid of HT patients and play important roles in lymphocyte recruitment and TLSs formation, providing key insight into the mechanisms underlying the pathogenesis of HT [163].

TLSs critically influence autoimmune pathogenesis through immune activation, autoantibody production, and inflammatory maintenance: Synovial TLSs in RA amplify local inflammation and autoantibody production; Salivary gland TLSs in SS drive class‐switch recombination; Renal TLSs in SLE, LN, and IgA nephropathy sustain tissue‐specific immunity, while TLSs in MS propagate cytokine‐mediated neurodegeneration. Mesenteric/creeping fat TLSs in CD trigger TNF‐α‐linked lymphatic dysfunction to exacerbate intestinal injury; rare islet TLSs in human T1D (abundant in NOD mice) promote β cell destruction via autoreactive T cells; thyroid TLSs in HT, guided by CCL21+ stromal cells, fuel anti‐TPO/TG autoantibody production and thyrocyte loss.

### 5.2 Relationship Between TLSs and Cancer

The presence and characteristics of TLSs within the tumor microenvironment are increasingly recognized as critical determinants of tumor progression, anti‐tumor immunity, and clinical outcomes. The studies of TLSs not only deepen our understanding of cancer immunobiology but also uncover potential ways for new therapeutic strategies [1, 238, 239]. The occurrence of TLSs can vary depending on the type of cancer, and TLSs have been identified in a variety of human cancers, including NSCLC, CRC, ovarian cancer, and melanoma [33, 114, 240, 241, 242]. The prognostic significance of TLSs is closely related to their anatomical location and maturation state [77, 243]. In most solid tumors, the presence of intra‐tumoral TLSs is consistently associated with a good prognosis and may reflect a robust and local anti‐tumor immune response [244, 245]. In contrast, the prognostic value of peri‐tumoral TLSs varies widely between cancer types. For instance, their presence correlates with improved survival in esophageal squamous cell carcinoma (ESCC) and CRC [173, 181], yet may hold neutral or even negative in others. In addition, the maturation status of TLSs, particularly the presence of GCs, serves as a strong positive prognostic indicator, underscoring the functional capacity of these structures in supporting adaptive immunity [116, 246]. In this section, we illustrate the associations between TLSs and tumor prognosis, highlighting their heterogeneity across various cancer types (Table 3).

#### 5.2.1 TLSs in NSCLC

Lung cancer, one of the most common cancers worldwide, originates from the cells within the lungs and is a major cause of cancer‐related deaths globally. It is broadly divided into two main types based on its origin and cellular characteristics: NSCLC and small cell lung cancer (SCLC). NSCLC, with the most common subtypes being lung adenocarcinoma (LUAD) and lung squamous cell carcinoma (LUSC), remains the major cause of cancer‐related deaths worldwide despite advances in diagnosis and therapy [168, 169]. Multiple studies underscore the vital role of TLSs in enhancing prognoses and therapeutic outcomes in NSCLC patients. It has been demonstrated that in NSCLC, the maturity and abundance of TLSs significantly affect the therapeutic effect of neoadjuvant chemotherapy (NCT), and the disease‐free survival (DFS) levels are improved in patients with higher TLS maturity and higher numbers [170]. This suggests that promoting the maturation of TLSs may be a new way to improve the efficacy of NCT.

In NSCLC, the presence of TLSs is a strong positive prognostic biomarker, consistently associated with improved survival outcomes and enhanced responses to immunotherapy. mTLSs containing GCs are frequently observed in NSCLC tissues and are most closely associated with prolonged patient survival, emphasizing the importance of a structured, functional niche for adaptive anti‐tumor immunity [28, 33]. The cellular composition of these structures is vital; TLSs rich in CD8^+^ cytotoxic T cells and CD4^+^ T helper cells are linked to potent effector function and tumor cell killing [33]. Furthermore, the role of B cells within TLSs is crucial because their presence and the production of tumor‐specific antibodies are important components of protective immune response and predict favorable clinical outcomes [4, 7]. From a therapeutic perspective, the density and maturity of TLSs have become key predictors of positive response to immune checkpoint blockade (e.g., anti‐PD‐1/PD‐L1 therapy) [36]. Patients with mTLSs in their tumors often exhibit increased infiltration of activated T cells and a microenvironment more conducive to reversing T‐cell exhaustion, resulting in superior treatment efficacy [35, 36]. Consequently, TLSs are not only prognostic markers but also promising biomarkers for guiding immunotherapy strategies in NSCLC, highlighting their translational significance in oncology.

#### 5.2.2 TLSs in CRC

CRC, a heterogeneous malignant disease, originating from the glandular tissues of the colon's mucosa, accounts for approximately 10% of all annually diagnosed cancers and cancer‐related deaths worldwide [247, 248]. In this context, TLSs have emerged as critical organizers of anti‐tumor immunity and are strongly associated with improved patient outcomes. The prognostic value of TLSs in CRC is closely linked to their anatomical location and maturation state. The presence of mature, intra‐tumoral TLSs consistently correlates with prolonged relapse‐free survival (RFS) and OS, underscoring their role in sustaining a potent, localized anti‐tumor response [171, 172]. Conversely, the significance of peri‐tumoral TLSs is more complex and may depend on other microenvironmental factors, such as tumor stroma percentage [172, 173].

Mechanistically, the formation and function of TLSs in CRC are driven by a coordinated network of cells and chemokines. B cells are the main component of infiltrating immune cells in CRC [174]. Beyond producing anti‐tumor antibodies (the TLS‐associated mAb6 that inhibits tumor growth in models), B cells contribute to TLS formation and T cell activation through antigen presentation [94, 174]. Their recruitment and organization are facilitated by specific chemokine axes: fibroblast‐derived CCL19 promotes B cell infiltration and TLS formation, especially in the metastatic settings, while CXCL13‐producing CD8^+^ T cells play a key role in the initiation of TLS structure [94, 174]. Furthermore, the CCL28–CCR10 axis guides IgG plasma cells to strategically localize from the TLS periphery to the tumor stroma to achieve effective antibody‐mediated immunity [174]. Consequently, patients whose tumors are enriched with these TLS and B cell signatures have significantly improved responses to immunotherapy, highlighting the translational significance of these structures.

#### 5.2.3 TLSs in Ovarian Cancer

Ovarian cancer is a major malignancy of the female reproductive system, which is broadly divided into epithelial ovarian cancer, stromal tumors, and germ cell tumors according to its cell origin. Epithelial ovarian cancer accounts for about 90% of cases, among which high‐grade serous ovarian cancer (HGSOC) is the most frequent histological subtype [175]. In HGSOC, the presence of TLSs supports B cell maturation, facilitates immunoglobulin production, and enhances T cell–B cell interactions [175, 176]. However, unlike observations in NSCLC, mTLSs are infrequent in HGSOC, developing in only a minority of tumors with high TMB, these structures are not only rare but also exhibit strong immunosuppressive features, correlating with bad prognosis [153]. At the cellular level, PD1^+^CD8^+^ T cells within HGSOC‐associated mTLSs lack the TCF1^+^ phenotype indicating immune checkpoint blockade (ICB) sensitivity; instead, they mainly display a TIM3^+^ phenotype linked to ICB resistance [177, 178].

Notably, though some studies associate TLSs presence with improved outcomes in ovarian cancer, this benefit appears to depend on the context. For instance, CDK4/6 inhibitors (CDK4/6i) have been shown to promote TLSs formation and enhance the efficacy of anti‐PD1 therapy, possibly through mechanisms involving SCD1 regulation and downstream mediators such as ATF3 and CCL4 [238]. Overall, the presence of TLSs in ovarian cancer—particularly their maturity, spatial distribution, and cellular composition—has important prognostic and therapeutic implications. Current evidence indicates that HGSOC has a series of immune aggregates in different tissues, among which mTLSs are rare and correlate with an immune environment dominated by TIM3^+^PD1^+^ CD8^+^ T cells, and TFH cells are relatively scarce. This microenvironment may be the foundation for the subtype's generally limited response to ICIs [153].

In short, TLSs in ovarian cancer are not simply good or bad—it depends on the subtype, maturity, and context. Strategies aimed at inducing or therapeutically treating the utilization of TLSs hold promise for improving immunotherapy responses and clinical outcomes in some settings, but their potential as biomarkers and targets must be weighed against the negative role seen in HGSOC.

#### 5.2.4 TLSs in Esophageal Cancer

Esophageal cancer (EC) is the seventh most commonly diagnosed cancer and the fifth leading cause of cancer‐related mortality worldwide. The two main tissue subtypes are ESCC and esophageal adenocarcinoma (EAC). Recent studies in ESCC have indicated that the presence of mTLSs is associated with improved survival in patients undergoing surgery alone (SA) [79, 179, 180, 181] or NCT [185]. Notably, the presence of mTLSs itself has been shown to be a more robust prognostic indicator than mTLS density, suggesting that evaluating mTLS presence is a highly effective method for predicting clinical outcomes in ESCC patients, regardless of neoadjuvant treatment status [182].

Furthermore, the distribution, abundance, and maturity of TLSs are closely linked to OS in ESCC. Specifically, the abundance of peritumoral TLSs has been identified as an independent prognostic factor for OS [185]. Interestingly, the prognostic impact of TLS abundance varies by tissue compartment: high densities of intratumoral TLSs and TLSs at the edge of invasion are associated with better outcomes, whereas abundant peritumoral TLSs correlate with worse prognosis. Moreover, the cellular composition of mTLSs—especially the presence of proliferative and activated CD20^+^Ki‐67^+^ B cells, CD21^+^ DCs, and CD4^+^Ki‐67^+^ T helper cells—significantly influences ESCC prognosis [185].

TLSs, which are present in various malignancies including ESCC, serve as active hubs of humoral immunity in the tumor microenvironment. They facilitate the production of diverse immunoglobulin (Ig) subtypes that exert distinct functional effects. Differences in immunoglobulin expression in TLSs can modulate their overall role in the tumor microenvironment [183]. Beyond TLS quantity, the immune heterogeneity in TLSs may be essential for personalized assessment of tumor immunotherapy efficacy. Both TLSs and tumor‐infiltrating lymphocytes (TILs) not only have prognostic correlations in ESCC but also exhibit significant spatial heterogeneity [184].

Collectively, these findings emphasize the clinical importance of evaluating the tumor microenvironment, particularly TLSs in the management and prognostic stratification of ESCC. This highlights the vital role of TLSs in shaping cancer progression and therapeutic response.

#### 5.2.5 TLSs in Other Cancers

In addition to the cancers previously mentioned, TLSs have been identified in a wide range of malignancies, and their density, maturity, and spatial organization are increasingly recognized as key determinants of prognosis and therapeutic response.

Existing evidence emphasizes that the presence of mTLSs containing GCs, correlates with better clinical outcomes than early TLSs without organized GCs. This association has been consistently observed in hepatocellular carcinoma (HCC) [8, 249], lung squamous cell carcinoma [75], CRC [171], and pancreatic cancer [250]. Furthermore, the indicative transcriptional signatures of B cells and PCs, coupled with high PC densities, are linked to longer survival in multiple cancer types [251]. Notably, the presence of B cells and mTLSs tends to predict response to ICIs and OS more accurately than T cell markers alone, a finding confirmed in soft tissue sarcoma [7, 36], melanoma [4, 252], renal cell cancer [4, 36], urothelial cancer [36, 253], and other cancer types [36]. The specific mechanisms by which B cells and PCs play these beneficial effects remain an active area of research [246, 251].

The spatial relationship between TLSs and tumor cells has become an important factor affecting immune function and treatment response. In recurrent/metastatic head and neck squamous cell carcinoma (R/M HNSCC), the closer the TLSs are to tumor cells, the more positive the response to ICIs, indicating that effective anti‐tumor immunity requires close lymphoid–tumor interactions [186]. This principle is reinforced in muscle‐invasive bladder cancer, where TLSs are located near the front edge of tumor invasion, correlated with inflammatory immune phenotype and improved patient survival [187]. The spatial structure thus provides critical insights into differences in immunotherapies efficacy.

In thyroid cancer, particularly anaplastic thyroid cancer (ATC), enrichment of CXCL13^+^ T lymphocytes is implicated in the development of early TLSs, which may make these tumors more sensitive to combination immunotherapy such as famitinib and anti‐PD‐1 antibodies [254]. Studies of specific cancer types reveal unique aspects of TLS biology. In HCC, intratumoral TLSs density is high after neoadjuvant immunotherapy, which is associated with significant pathologic response and improvement of RFS. Interestingly, areas of tumor regression exhibit involuted morphology of TLSs with dispersed B cell follicles but persistent T cell zones rich in T cell‐dendritic cell interactions and memory T cell markers, suggesting that late‐stage TLSs play a role in maintaining T cell memory post‐therapy [255]. In HNSCC, mTLSs are enriched with stem‐like T cells and B cells at various maturation stages, with progenitor exhausted CD4^+^ T cells playing a key role in coordinating intra‐tumoral immunity [96]. Similarly, in breast cancer brain metastases (BCBM), the presence of intratumoral TLSs and higher TLS scores are positive prognostic indicators, linked to better overall and progression‐free survival [256].

Nevertheless, the role of TLSs remains underexplored in several common malignancies, including gastric cancer, prostate cancer, and pediatric solid tumors, where systematic TLS characterization is lacking. Moreover, even in well‐studied cancer types, the causal relationship between TLS formation and antitumor immunity has not been established. Future priorities should include: establishing standardized TLS assessment protocols applicable to rare cancers; conducting prospective cohort studies to validate TLS as a predictive biomarker across diverse treatment modalities; and investigating whether therapeutic TLS induction can synergize with existing immunotherapies in “cold” tumors.

In summary, the available data suggest that mTLSs are associated with improved antitumor immune response and survival in various cancers. However, it is unknown whether TLS formation is a consequence or a prerequisite for effective antitumor immunity. Future research to elucidate the causal drivers of TLS neogenesis and its functional maintenance in the tumor microenvironment will be crucial for therapeutic utilization of these structures.

## 6 The Potential Significance of TLSs in Disease Diagnosis and Surveillance

TLSs demonstrate significant potential as dual‐purpose biomarkers for both prognostic stratification and therapeutic monitoring in oncology, critically influencing antitumor immunity and therapeutic efficacy [47, 257, 258, 259]. Emerging evidence supports their utility in predicting immunotherapeutic responsiveness [141], with TLSs density and maturation status reflecting dynamic disease activity and progression trajectories. In autoimmune pathologies such as RA and pSS, TLSs burden correlates with clinical severity, offering a framework for personalized therapeutic strategies. Therapeutically, TLSs modulation represents a promising intervention target to attenuate chronic inflammation through localized immune regulation, while also holding translational value in enhancing tumor immunotherapy. Beyond autoimmune and oncological contexts, TLSs may contribute to pathogen clearance and adaptive immune memory in infectious diseases, though their spatiotemporal regulation requires further mechanistic elucidation [162, 260, 261]. Clinically, the potential significance of TLSs in disease diagnosis and monitoring is primarily evidenced in several key areas (Figure 2).

## 7 Treatment Strategies and Prospects for TLSs

TLSs represent a promising therapeutic target in autoimmune disease management. Current TLSs detection methodologies span conventional histopathological techniques (e.g., IHC, IF) and advanced modalities such as imaging mass cytometry (IMC) [262, 263], which provides high‐dimensional spatial resolution for analyzing TLSs cellular architecture. Therapeutic interventions focus on immunomodulatory agents targeting TLS‐resident lymphocytes, including B cell‐depleting therapies (e.g., rituximab against CD20^+^ B cells in autoimmune diseases and lymphomas) [35, 264, 265, 266], TNF‐α inhibitors (infliximab, adalimumab) to disrupt TLSs functionality in RA, and small‐molecule inhibitors of signaling pathways like Janus kinase (JAK)/STAT [267, 268]. Adjunctive anti‐inflammatory agents, notably corticosteroids and NSAIDs, suppress TLS‐associated inflammatory cascades.

Potential future research directions and therapeutic strategies for TLSs include the following aspects (Table 4). At first, targeting key molecules involved in the formation and maintenance of TLS, which involves investigating critical molecules such as CXCL13 [269, 270], CXCL4, and CD40/CD40L, was used to develop corresponding antagonists or agonists to modulate TLSs function. In addition, CAR‐T cell therapy [271, 272], as a form of cell therapy, enhances anti‐tumor immune responses by selectively targeting specific cell populations within TLSs, particularly those localized in the tumor microenvironment. Furthermore, implementing vaccination strategies may stimulate the immune response within TLSs and augment the immune response against pathogens, with particular emphasis on infectious diseases and cancer therapy [273, 274]. Moreover, the investigation and development of immune checkpoint molecules within TLSs, such as PD‐1/PD‐L1 in the field of immune checkpoint inhibitor research [275, 276, 277], could enhance localized immune responses. In addition, in terms of microenvironment regulation, examine the microenvironment of TLSs, including factors such as oxygen supply, metabolites, ECM, and devise therapeutic strategies to modulate the microenvironment to influence TLSs’ function. In field of gene therapy, employing gene editing methodologies, notably CRISPR/Cas9 [278, 279], to directly alter gene expression within cells in TLSs for the treatment of both inherited and acquired diseases. And studies have shown that the cyclic GMP‐AMP synthase (cGAS) pathway of the stimulator of interferon genes (STING) augments humoral immunity and antitumor responses [280, 281].

**TABLE 4:** Treatment strategies and prospects for TLSs.

Therapies	Prospects and role	Predictive value	Refs.
Targeted therapy	Study of key molecules involved in TLS formation and maintenance, such as CXCL13, CXCL4, and CD40/CD40L, and development of corresponding antagonists or agonists to modulate TLSs function.	Positive	[270, 271]
Cell therapy	CAR‐T cell therapy technology is used to directly target specific cell populations in TLS, particularly in the tumor microenvironment, to enhance anti‐tumor immune responses.	Positive	[272, 273]
Vaccination	Implement vaccination strategies to stimulate the immune response within TLS and enhance the immune response to pathogens.	Positive	[274, 275]
Immune checkpoint inhibitors	Research and development of immune checkpoint molecules in TLS, such as PD‐1/PD‐L1, to enhance local immune responses.	Positive	[276, 277, 278]
Gene therapy	Gene editing methods, specifically CRISPR/Cas9, directly alter gene expression within TLS cells for the treatment of inherited and acquired diseases.	Positive	[279, 280]
The circular AMP (cGAS) pathway of the interferon gene (STING)	Enhances humoral immunity and antitumor responses.	Positive	[281, 282]
Microenvironment regulation	Examine the microenvironment of TLSs, including factors such as oxygen supply, metabolites, and the extracellular matrix, and devise therapeutic strategies to modulate the microenvironment to influence TLSs function.	Positive	—

In summary, therapeutic strategies targeting TLSs are currently undergoing rapid development. Future research will increasingly focus on elucidating the mechanisms underlying TLSs formation and function, as well as on the innovation of novel treatments that can more effectively modulate immune responses and address disease.

## 8 Clinical Translation: Preclinical and Therapeutic Insights

Given the clear value of TLSs in predicting disease outcomes, treatments that aim to induce or modulate these structures have become a promising approach for both

 autoimmune diseases and cancer. These strategies mainly include: targeting key chemokine signals like CXCL13‐CXCR5 needed for TLS formation; blocking or activating specific pathways like LTβR, ICOS inside TLSs; and using TLSs as a marker to help select patients for current immunotherapies. The following section synthesizes the main directions and research status in this field, with key preclinical and clinical studies summarized in Table 5.

**TABLE 5:** Summary of selected preclinical and clinical studies targeting TLS formation or function.

	Target/Mechanism of action	Agent/Therapy	Disease context	Key finding/Effect on TLS	Current stage	Refs./Trial ID
I. Modulating chemokine axes for TLS induction	CXCL13‐CXCR5 axis	Anti‐CXCL13 mAb/CXCR5 inhibitors	RA, SLE (preclinical); Solid Tumors (exploratory)	Blockade inhibits TLS formation and germinal center reactions; reduces autoantibody titers and disease severity.	Preclinical	[31, 140, 143, 144, 145]
	CCL19/CCL21‐CCR7 axis	Recombinant CCL19/21; CCR7 agonists	Cancer	Ectopic expression promotes lymphocyte aggregation and TLS neogenesis, enhancing anti‐tumor immunity.	Preclinical	[31, 140, 143, 144, 145]
II. Targeting lymphoid organization and cell survival	LTβR signaling	LTβR agonists	Cold Tumors (e.g., pancreatic cancer models)	Agonism induces de novo TLS formation, remodels TME, and sensitizes tumors to checkpoint blockade.	Preclinical/Early Clinical	[28, 33, 35, 36, 169, 170, 171]
	ICOS‐ICOSL costimulation	Anti‐ICOS agonists	Solid Tumors (clinical trials)	Promotes Tfh cell expansion and TLS maturation within tumors; under evaluation in combo with anti‐PD‐1.	Phase I/II	[221, 222, 223]
III. TLS as a predictive biomarker for immunotherapy	PD‐1/PD‐L1 + TLS status	Pembrolizumab, Atezolizumab, etc.	NSCLC, STS, TNBC, HNSCC	Presence of mature TLSs enriches for patients with superior objective response rates (ORR) and survival to ICIs.	Approved/Late‐phase Trials	NCT04095208, NCT04705818, NCT06313463
	PD‐1/CTLA‐4 bispecific + TLS	MEDI5752 (PD‐1/CTLA‐4 bispecific)	Advanced Solid Tumors with mTLS	Selectively enrolling patients with mature TLS to evaluate enhanced efficacy of dual checkpoint blockade.	Phase II (Recruiting)	NCT05888857
	Combination ICI based on TLS	Atezolizumab ± Tiragolumab (anti‐TIGIT)	Metastatic Lung Cancer	Randomizing TLS+ patients to evaluate if adding TIGIT blockade to PD‐L1 inhibition improves outcomes.	Phase II (Not Yet Recruiting)	NCT06713798
	Chemotherapy + ICI in TLS+ patients	Camrelizumab (anti‐PD‐1) + Capecitabine	TNBC (non‐pCR, TLS+)	Adjuvant combo therapy vs. chemo alone in TLS+ patients to improve DFS.	Phase III (Recruiting)	NCT06313463
IV. Novel therapeutic combinations	MDM2‐p53 + PD‐1	MDM2i + Ezabenlimab (anti‐PD‐1)	STS, NSCLC, TNBC, etc. (TLS+)	Combining p53 activation with PD‐1 blockade in TLS+ tumors to overcome resistance.	Phase II (Withdrawn)	NCT06084689
V. Observational and diagnostic studies	TLS as a Prognostic classifier	TLS scoring via digital pathology	Gastric Cancer, NPC, Prostate Cancer, RCC, HCC	High TLS density/maturity independently predicts longer OS, DFS; guides risk stratification.	Completed/Recruiting	NCT06979817, NCT06770699, NCT06484127, NCT06883565, NCT06542796
	TLS in autoimmunity	Tissue analysis	cGVHD, Autoimmune Bullous Diseases	Characterizing TLS presence in target tissues to understand pathogenesis.	Recruiting/Completed	NCT0247150, NCT04509570

Data sources: https://clinicaltrials.gov/

## 9 Summary and Outlook

TLSs have emerged as important and context‐dependent regulators of immune responses, bridging autoimmune diseases and cancer through common inflammatory origins and different functional roles that define their “double‐edged sword” effect. This review systematically synthesizes core findings across TLS biology: structurally, TLSs resemble secondary lymphoid organs with organized zones for different immune cells, specialized stromal networks, and blood vessels that facilitate lymphocyte recruitment, supported by conserved pathways driving their inflammation‐induced formation. Functionally, TLSs have opposite roles. On one hand, in autoimmune diseases, they are harmful engines that drive autoreactive immunity and tissue damage, leading to worse prognosis. On the other hand, in most cancers, they are beneficial structures that enhance anti‐tumor immunity, resulting in better survival and treatment response. Clinically, TLSs show great promise as biomarkers for both diagnostic and predicting disease outcomes. Overall, the evidence highlights that TLSs are key conductors of abnormal local immune responses, with their function being entirely determined by the surrounding tissue and disease context.

Despite significant advances in characterizing TLSs, critical knowledge gaps persist. First, the specific signals that trigger TLS formation and maturation in different tissues are still not fully understood. The starting signals and cell interactions that drive TLS development vary from one disease to another and need more detailed study. Second, it is still unclear how TLSs functionally adapt between being pathogenic or regulatory, and what key checkpoints control this transition. Third, standardized methods for TLS detection and quantification are still missing. Advanced techniques exist, but turning TLSs into a routine clinical tool requires consistent application across institutions and validation in large‐scale studies. Finally, a key challenge is that animal models often don't fully mirror human disease. Therefore, more accurate models are needed to properly evaluate therapies that target TLSs.

Although TLSs have been identified in many autoimmune diseases and cancers, the exact molecular mechanisms of their formation remain poorly understood. This lack of clarity poses a significant challenge to the study of the specific mechanisms by which TLS plays a role in autoimmune diseases and cancers, complicating their use as prognostic indicators in different autoimmune diseases and cancers. Future research should focus on investigating the mechanism of the formation and maintenance of TLSs, the relationship between TLSs and disease progression and prognosis, and the treatment strategies targeting TLSs in autoimmune diseases and cancers against TLSs. In addition, therapeutic development should exploit this functional duality. For autoimmune diseases, the goal is to block the formation or disrupt the function of harmful TLSs to reduce local immune attack and tissue damage. For cancer, the aim is the opposite: to promote the growth of mTLSs or combine TLS‐focused treatments with existing immunotherapies, in order to boost anti‐tumor immunity and treatment outcomes.

In conclusion, TLSs are an indispensable multifaceted component of the immune response, and comprehensive studies of their function and mechanisms are essential to elucidate their role in autoimmune diseases and cancers and to establish a scientific basis for the development of new therapeutic strategies. By deepening our understanding of their molecular mechanisms, functional heterogeneity, and clinical relevance, we can develop precision strategies that use TLSs to mitigate pathogenic immunity in autoimmunity and strengthen anti‐tumor responses in cancer.

## Author Contributions

G.Y. drafted the manuscript. G.Y., G.L., and M.L. revised the manuscript. G.Y. and M.L. visualized the data. G.Y., D.Y., C.D., and X.T. investigated the study. G.L., M.L., and Y.X. supervised the study. M.L. and Y.X. conceptualized the study. Y.X. acquired funding. All authors reviewed the manuscript.

## Ethics Statement

The authors have nothing to report.

## Conflicts of Interest

The authors declare no conflicts of interest.

## Data Availability

The authors have nothing to report.
